# Trends in Stroke Burden and Rehabilitation Demand in Saudi Arabia, 1990–2021, with Projections to 2030: A National Analysis Using GBD 2021 Data

**DOI:** 10.3390/jcm15062382

**Published:** 2026-03-20

**Authors:** Faisal Alenzy, Saleh A. Abu Araigah, Maha Almarwani, Vishal Vennu, Saad M. Bindawas

**Affiliations:** 1 Neuro-Rehabilitation Unit, Physical Therapy Department, Prince Sultan Military Medical City (PSMMC), Riyadh 12233, Saudi Arabia; 2 Department of Rehabilitation Sciences, College of Applied Medical Sciences, King Saud University, Riyadh 11433, Saudi Arabia

**Keywords:** stroke, years lived with disability, years of life lost, rehabilitation, Global Burden of Disease, Saudi Arabia, health system planning, subarachnoid hemorrhage, intracerebral hemorrhage, Vision 2030

## Abstract

**Background/Objectives**: Stroke is a leading cause of mortality and disability in Saudi Arabia; however, national estimates of stroke-related rehabilitation needs remain limited. This study quantified temporal trends in stroke incidence, prevalence, premature mortality, and disability from 1990 to 2021. It also examined disparities in stroke-related disability by subtype, sex, and age in 2021 and projected rehabilitation demand to 2030 to inform health system planning under Vision 2030. **Methods**: We conducted a secondary analysis of Global Burden of Disease (GBD) 2021 estimates for Saudi Arabia. Age-standardized rates for incidence, prevalence, years of life lost (YLLs), and years lived with disability (YLDs) were extracted for overall stroke and three subtypes: ischemic stroke, intracerebral hemorrhage (ICH), and subarachnoid hemorrhage (SAH). Temporal trends were evaluated using log-linear regression to estimate the average annual percentage change (AAPC). YLDs were mapped to severity levels and four rehabilitation modalities, physiotherapy (PT), occupational therapy (OT), speech–language therapy (SLT), and multidisciplinary comprehensive rehabilitation (MCR), using utilization probabilities informed by the literature. Projections to 2030 incorporated national population forecasts and included 95% prediction intervals and sensitivity analyses. **Results**: From 1990 to 2021, age-standardized stroke incidence declined from 166.3 to 130.7 per 100,000 (−21.4%; AAPC, −0.86%, *p* = 0.004), prevalence from 982.4 to 965.2 per 100,000 (−1.8%; AAPC, −0.10%, *p* = 0.056), and YLL rates from 3209.0 to 1893.4 per 100,000 (−41.0%; AAPC, −1.76%, *p* < 0.001). In contrast, YLD rates declined modestly from 133.5 to 129.9 per 100,000 (−2.7%; AAPC, −0.13%; *p* = 0.032). Despite these reductions in age-standardized rates, absolute stroke-related YLDs more than tripled, increasing from approximately 10,900 (95% UI: 8100–13,900) in 1990 to 36,245 (95% UI: 26,600–46,100) in 2021, largely driven by population growth and aging. In 2021, ischemic stroke accounted for 71.1% of total YLDs, followed by ICH (20.3%) and SAH (8.5%). Among adults aged 15–49 years, females had higher hemorrhagic YLD rates than males, with particularly pronounced differences for SAH (female-to-male ratio, 1.5–1.7). By 2030, the projected YLD-equivalent workload, a standardized proxy measure of relative service demand rather than a direct headcount of required therapists, is expected to increase to 29,758 for PT, 21,809 for OT, 14,879 for SLT, and 15,083 for MCR. Sensitivity analyses showed that rehabilitation demand estimates were sensitive to assumptions regarding severity distribution, with a hemorrhagic-weighted scenario increasing projected MCR demand by 6.8%. **Conclusions**: The increasing absolute burden of stroke-related disability in Saudi Arabia, despite declining age-standardized rates and substantial reductions in premature mortality, highlights the necessity to expand rehabilitation capacity. Scaling community-based, outpatient, and telerehabilitation services in alignment with the Health Sector Transformation Program and integrating disability-informed planning into Vision 2030 should be prioritized.

## 1. Introduction

Stroke is a leading cause of mortality and long-term disability worldwide [1,2]. It contributes substantially to years lived with disability (YLDs) and places considerable demand on rehabilitation services. Global Burden of Disease (GBD) estimates indicate that, between 1990 and 2021, the absolute numbers of incident strokes, prevalent cases, and disability-adjusted life years (DALYs) increased [3]. This increase occurred despite declining age-standardized rates and was driven primarily by population growth and ageing [4]. Improvements in post-stroke survival have further heightened the need for long-term rehabilitation. Consequently, stroke has become a major contributor to the global burden of rehabilitation services [5,6].

In Saudi Arabia, the burden of stroke is increasing within a rapidly evolving demographic and health system context. The population has expanded substantially, urbanization has accelerated, and a growing proportion of individuals are surviving to older age. National studies have documented an increasing stroke burden and projected further growth in the coming decades [7,8]. Previous epidemiological modelling in Saudi Arabia has focused primarily on first-ever stroke incidence to inform acute care planning [9]. Rehabilitation-focused studies have examined implementation frameworks [10], functional outcomes following inpatient rehabilitation [11], and medical complications during rehabilitation [12]. However, these investigations have not quantified population-level disability using standardized YLD metrics or translated disability estimates into projections of future rehabilitation demand.

Ongoing health system transformation efforts further underscore this gap. Saudi Arabia’s Vision 2030 and the Health Sector Transformation Program emphasize value-based care, integrated clinical pathways, and expansion of rehabilitation services [13,14,15,16,17]. Despite these initiatives, the Saudi Stroke Standards continue to identify persistent limitations in both inpatient and community-based rehabilitation. Deficiencies in early supported discharge and continuity of post-acute care remain evident [14,18,19,20]. Reliable, disability-informed projections are therefore essential for effective service planning.

Using GBD 2021 estimates, this study describes long-term trends in stroke incidence, prevalence, premature mortality (YLLs), and YLDs from 1990 to 2021. It further examines disparities in stroke-related YLDs by subtype, including the distinct contributions of ischemic stroke, intracerebral hemorrhage (ICH), and subarachnoid hemorrhage (SAH), as well as differences by sex and age group in 2021. Finally, the study projects stroke-related disability and rehabilitation demand to 2030, stratified by severity level and rehabilitation modality.

The primary objective of this study is descriptive and projection-based. It seeks to quantify temporal trends in stroke-related disability and to generate illustrative projections of rehabilitation demand to inform strategic health system planning. The study does not aim to provide precise workforce estimates or causal forecasts. Rather, it translates population-level YLD estimates into structured rehabilitation demand scenarios aligned with Saudi Arabia’s Vision 2030 priorities.

## 2. Materials and Methods

### 2.1. Study Design

This study is a secondary analysis of modeled epidemiological estimates from the GBD 2021 study, combined with national population data from the General Authority for Statistics (GASTAT) of Saudi Arabia. Because the analysis was based on secondary, model-derived estimates rather than primary observational data, the Strengthening the Reporting of Observational Studies in Epidemiology (STROBE) guidelines were applied selectively to relevant reporting components, with acknowledgment of their limited applicability [21]. Reporting primarily adhered to the Guidelines for Accurate and Transparent Health Estimates Reporting (GATHER), which are specifically designed for studies based on modeled health estimates [22] (Supplementary Tables S1 and S2). This approach is consistent with reporting practices adopted in previous national GBD-based analyses, including country-level burden assessments such as the GBD 1990–2021 oral disorders study in China [23].

### 2.2. Data Sources

#### 2.2.1. GBD 2021 Stroke Estimates

GBD 2021 provides internally consistent estimates of incidence, prevalence, mortality, years lived with disability (YLDs), years of life lost (YLLs), and disability-adjusted life years (DALYs) for stroke and other conditions by country, age, sex, and year, using standardized modeling methods [1,2,3,4]. For this study, country-level estimates for Saudi Arabia were obtained from the Institute for Health Metrics and Evaluation (IHME) GBD 2021 Results Tool and associated downloadable data files [24]. Estimates were extracted for seven milestone years: 1990, 1995, 2000, 2005, 2010, 2015, and 2021. The following measures were extracted: incident stroke cases (counts and age-standardized rates per 100,000); prevalent stroke cases (counts and age-standardized rates per 100,000); years of life lost due to premature mortality (YLLs; counts and age-standardized rates per 100,000); years lived with disability (YLDs; counts and age-standardized rates per 100,000); and disability-adjusted life years (DALYs; counts and age-standardized rates per 100,000).

Estimates were obtained separately for total stroke, ischemic stroke, intracerebral hemorrhage (ICH), and subarachnoid hemorrhage (SAH). All GBD estimates include 95% uncertainty intervals (UIs), which reflect propagated uncertainty arising from input data sources, modeling assumptions, and parameter estimation [1,2,3]. Age-specific estimates were extracted for the following groups, 10–19, 20–24, 25–29, 30–34, 35–39, 40–44, 45–49, 50–54, 55–59, 60–64, 65–74, and 75 years and older, in addition to the age-standardized rate.

#### 2.2.2. Case Definition and Coding

Stroke was defined according to the GBD nonfatal disease hierarchy, which operationalizes stroke using standardized case definitions aligned with International Classification of Diseases (ICD) codes. The GBD framework maps ICD-10 codes for ischemic stroke (e.g., I63.x), intracerebral hemorrhage (I61.x), and subarachnoid hemorrhage (I60.x), along with corresponding ICD-9 codes, into mutually exclusive stroke subcategories [1,2,3]. Nonspecific cerebrovascular codes (e.g., I64) are redistributed across stroke subtypes within the GBD modeling framework using cause- and diagnostic-pattern-based approaches [3,24].

No modifications to case definitions or additional reclassification procedures were undertaken. All analyses reflect the GBD 2021 definitions and coding for the three stroke subtypes and total stroke.

#### 2.2.3. Population Data

Population counts and age distributions for Saudi Arabia were obtained from the General Authority for Statistics (GASTAT) and from the population input files used in GBD 2021 [24]. For projections to 2030, national population projections from GASTAT were used to convert projected age-standardized YLD rates into absolute YLD counts.

### 2.3. Measures and Metrics

Primary measures included age-standardized stroke incidence rates per 100,000 population (1990–2021); age-standardized prevalence rates per 100,000; age-standardized YLL rates per 100,000, used as a validated proxy for the burden of premature stroke mortality; age-standardized YLD rates per 100,000; and age-standardized DALY rates per 100,000.

All age-standardized rates were standardized to the GBD reference population age structure using GBD age-standardization weights. Rates were not standardized by sex; instead, sex-specific estimates were reported separately for males and females to characterize sex-based disparities.

Secondary measures included subtype-specific and sex-specific YLD rates and counts for 2021, stratified by age group (15–49 years, 50–69 years, and ≥70 years). These age categories follow standard GBD groupings, enabling comparability with prior national and international analyses. YLDs were used as a proxy for stroke-related disability and rehabilitation need, consistent with previous global rehabilitation modeling studies [5,6]. YLLs were included to capture the contribution of premature mortality and to characterize temporal divergence between mortality and disability.

### 2.4. Trend Analysis

Temporal trends in age-standardized rates from 1990 to 2021 were assessed using log-linear regression models of the form ln(Rateₜ) = β0 + β1 × t, where t denotes calendar year. The average annual percentage change (AAPC) was calculated as (e^β1^ − 1) × 100, with 95% confidence intervals (CIs) derived from the standard error of the slope coefficient. Models were fitted to seven GBD milestone-year observations (1990, 1995, 2000, 2005, 2010, 2015, and 2021), yielding five degrees of freedom per model. The limited number of time points may yield wider confidence intervals than analyses based on annually interpolated GBD data; this limitation was taken into account in the interpretation.

Standard regression diagnostics were applied to assess model fit and residual patterns. For interpretation, *p*-values < 0.05 were considered indicative of statistically significant trends, while recognizing that GBD rates and uncertainty intervals incorporate substantial modeled uncertainty.

Data management, statistical analysis, and visualization were performed using Python 3.10 (pandas, numpy, scipy, statsmodels, matplotlib) and Microsoft Excel 365. Trend modeling and forecasting were conducted using Python-based linear regression modules.

### 2.5. Disparity Analysis by Subtype, Sex, and Age

To characterize disparities in stroke-related disability in 2021, subtype-specific YLD rates and counts were summarized by sex and age group (15–49 years, 50–69 years, and ≥70 years). Relative differences in YLD rates were calculated between males and females and across age groups, and subgroups with disproportionately high YLD rates relative to their population size were identified.

This descriptive analysis focused on the distinct contributions of intracerebral hemorrhage (ICH) and subarachnoid hemorrhage (SAH) among younger adults, as well as sex differences in YLD rates across age strata. Female-to-male rate ratios were calculated for each subtype and age group to quantify sex-based disparities, in view of previous reports suggesting increasing stroke incidence among Saudi females [7,8,9,13].

### 2.6. Projection of Stroke-Related YLDs and Rehabilitation Demand to 2030

#### 2.6.1. Projection of YLD Rates

Age-standardized stroke YLD rates were projected from 2022 to 2030 using simple linear regression models fitted to the seven milestone-year observations, with calendar year as the independent variable and YLD rate (per 100,000) as the dependent variable. Separate models were developed for total stroke and for each stroke subtype (ischemic stroke, ICH, and SAH). Projections are presented with 95% prediction intervals derived from the regression models to reflect statistical uncertainty.

These projections assume the continuation of recent linear trends in age-standardized YLD rates and do not explicitly account for potential changes in risk-factor prevalence, treatment coverage, health-system performance, or demographic structural shifts. Because the projections are based on age-standardized rates, which adjust for population age structure, they may underestimate absolute future demand in rapidly aging populations. In Saudi Arabia, the proportion of individuals aged ≥ 65 years is projected to approximately double between 2021 and 2030. Accordingly, these projections should be interpreted as illustrative continuation-of-trend scenarios representing a conservative lower bound, rather than forecasts with causal interpretation.

#### 2.6.2. Conversion to Absolute YLD Counts

Projected age-standardized YLD rates were converted to absolute YLD counts using projected national population sizes for each year from 2022 to 2030 and the GBD age-standardization weights. For interpretability in health service planning, projected annual absolute YLDs are presented for 2025 and 2030 as key milestone years.

### 2.7. Severity Distribution and Rehabilitation Modality Mapping

To translate YLDs into estimates of rehabilitation demand, total stroke YLDs were mapped to severity strata and rehabilitation modalities through a multistep process informed by published evidence and expert-derived assumptions.

#### 2.7.1. Severity Distribution

Stroke-related YLDs were assigned to three disability severity categories: mild (modified Rankin Scale [mRS] 0–2), moderate (mRS 3), and severe (mRS 4–5), with corresponding proportions of 30%, 50%, and 20%. These proportions are based on prior global rehabilitation modeling studies [5,6], World Health Organization rehabilitation frameworks, and regional evidence on functional outcomes in Saudi stroke cohorts [11,12,25,26]. Application of a uniform distribution across stroke subtypes and time periods enables national-level projections of rehabilitation demand; however, this approach may underestimate the greater disability burden typically associated with hemorrhagic stroke, which is recognized as a limitation.

Rehabilitation workload was estimated by linking YLDs to intervention types using literature-based utilization probabilities derived from regional studies of clinical practice and functional outcomes [10,25,26]. For example, Alatawi [10] described current physiotherapy (PT) practices in Saudi stroke rehabilitation. In contrast, Winstein et al. [25] and Rodríguez-Orozco et al. [26] reported global and guideline-based estimates of rehabilitation intensity and modality allocation. The resulting YLD-equivalent workload provides a proxy measure of population-level rehabilitation needs, while acknowledging that it does not capture individual variation in treatment uptake or adherence.

To assess the robustness of rehabilitation demand estimates, sensitivity analyses were conducted using two alternative scenarios, with results presented in Supplementary Table S3. In Scenario A (hemorrhagic-weighted severity), a modified severity distribution of 20% mild, 40% moderate, and 40% severe was applied to intracerebral hemorrhage (ICH) and subarachnoid hemorrhage (SAH), reflecting the higher disability burden typically associated with hemorrhagic stroke subtypes. In comparison, the base-case distribution (30%, 50%, and 20%) was retained for ischemic stroke. In Scenario B (variation in utilization probabilities), rehabilitation modality utilization probabilities were varied uniformly by ±15% across all modalities and severity levels. These scenarios evaluated the sensitivity of rehabilitation demand projections to two key modeling assumptions: the distribution of disability severity and modality-specific utilization rates.

Some previously cited references [11,12] describe functional outcomes rather than direct utilization probabilities; however, they are retained to support severity-to-rehabilitation mapping within the regional context. The assumption of a constant severity distribution across subtypes and time periods simplifies modeling but may not reflect real-world heterogeneity; the sensitivity analyses partially address this limitation.

#### 2.7.2. Rehabilitation Modalities and Utilization Probabilities

Four principal rehabilitation modalities relevant to stroke survivors were considered: (1) physiotherapy (PT); (2) occupational therapy (OT); (3) speech–language therapy (SLT); and (4) multidisciplinary comprehensive rehabilitation (MCR), defined as coordinated PT, OT, SLT, and medical or nursing care delivered through inpatient or intensive outpatient or home-based programs.

Based on international stroke rehabilitation guidelines and regional practice surveys [17,25,26], and consistent with expert consensus from Saudi rehabilitation practice studies [10,11,12,17,26], modality-specific utilization probabilities were assigned according to disability severity. For mild disability, utilization probabilities were 0.50 for PT, 0.25 for OT, 0.15 for SLT, and 0.10 for MCR. For moderate disability, corresponding probabilities were 0.80 for PT, 0.60 for OT, 0.40 for SLT, and 0.40 for MCR. For severe disability, probabilities were 0.90 for PT, 0.80 for OT, 0.60 for SLT, and 0.70 for MCR.

These probabilities represent the proportion of YLDs in each severity category that are assumed to generate demand for a given rehabilitation modality. For example, among individuals with moderate disability, 80% were assumed to require PT and 40% to require SLT. Although derived from international guidelines and regional practice data, these estimates have not been validated against Saudi-specific service utilization records; this limitation is addressed in Section 4.3.

#### 2.7.3. YLD-Equivalent Rehabilitation Workload

The YLD-equivalent workload for each rehabilitation modality was calculated by summing the product of the YLD count for each severity category and its corresponding utilization probability. These YLD-equivalent units provide a standardized proxy measure of relative rehabilitation workload across modalities and over time. They do not represent the number of therapy sessions, the number of full-time staff equivalents, or facility capacity requirements. Rather, the projections are intended to inform strategic planning by characterizing the relative scale and trajectory of rehabilitation needs, rather than to define specific operational resource allocations.

### 2.8. Ethics

This study used aggregated, de-identified, publicly available data from GBD 2021 and official national statistics. No individual-level data were accessed. In accordance with institutional and national policies, this type of secondary analysis does not require ethical approval or informed consent.

## 3. Results

### 3.1. Temporal Trends in Stroke Burden, 1990–2021

Between 1990 and 2021, the age-standardized incidence of stroke in Saudi Arabia declined from 166.3 per 100,000 (95% uncertainty interval [UI]: 152.0–182.3) to 130.7 per 100,000 (95% UI: 119.4–141.7), corresponding to a 21.4% relative reduction (average annual percent change [AAPC]: −0.86%; 95% CI: −1.28 to −0.44; *p* = 0.004). Age-standardized prevalence decreased modestly from 982.4 to 965.2 per 100,000 (−1.8%), although this trend did not reach statistical significance (AAPC: −0.10%; 95% CI: −0.20–0.00; *p* = 0.056). The age-standardized YLD rate declined marginally from 133.5 to 129.9 per 100,000 (−2.7%; AAPC: −0.13%; 95% CI: from −0.24 to −0.02; *p* = 0.032) (Table 1).

In contrast, the age-standardized YLL rate declined substantially, from 3209.0 per 100,000 (95% UI: 2507.5–4006.7) in 1990 to 1893.4 per 100,000 (95% UI: 1556.8–2314.4) in 2021, representing a 41.0% reduction (AAPC: −1.76%; 95% CI: from −2.07 to −1.45; *p* < 0.001). Age-standardized disability-adjusted life year (DALY) rates decreased in parallel, from 3342.4 to 2023.3 per 100,000 (−39.5%; AAPC: −1.67%; 95% CI: from −1.97 to −1.38; *p* < 0.001). The magnitude of decline in premature mortality exceeded that observed for disability, resulting in a widening gap between mortality and disability trends over time.

**Figure 1:**
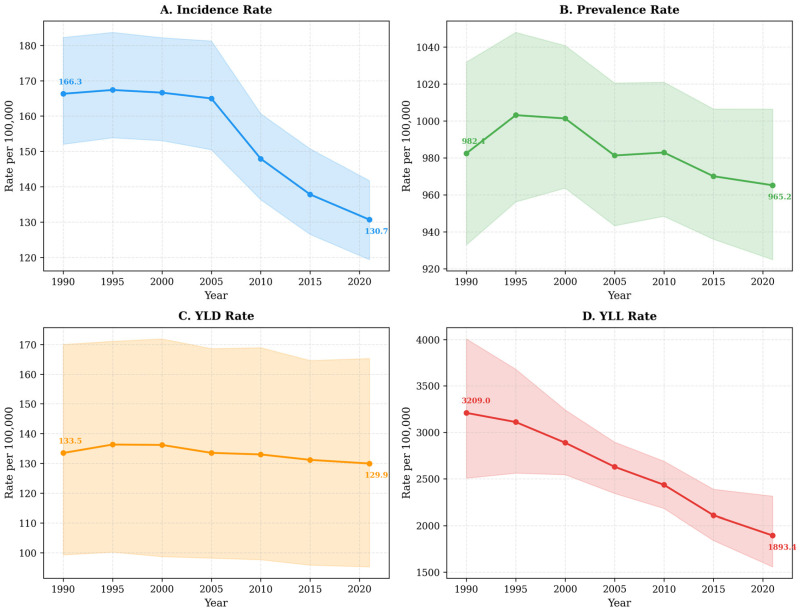
Age-standardized stroke burden in Saudi Arabia, 1990–2021. Panel (**A**): Incidence rate per 100,000. Panel (**B**): Prevalence rate per 100,000. Panel (**C**): YLD rate per 100,000. Panel (**D**): YLL rate per 100,000. Shaded areas represent 95% uncertainty intervals—estimates from the GBD 2021 study, combined for both sexes.

At the subtype level, the modest overall decline in YLD rates was attributable to reductions in hemorrhagic stroke (Supplementary Table S5). Age-standardized YLD rates for intracerebral hemorrhage decreased by 19.8%, from 25.2 to 20.2 per 100,000 (AAPC: −0.79%; *p* < 0.001), and for subarachnoid hemorrhage by 11.1%, from 9.4 to 8.3 per 100,000 (AAPC: −0.39%; *p* = 0.002). In contrast, the age-standardized YLD rate for ischemic stroke, which accounted for 78.0% of total stroke YLDs, remained stable over the study period, increasing non-significantly from 98.9 to 101.4 per 100,000 (+2.5%; AAPC: +0.05%; *p* = 0.323) (Figure 1).

**Figure 2:**
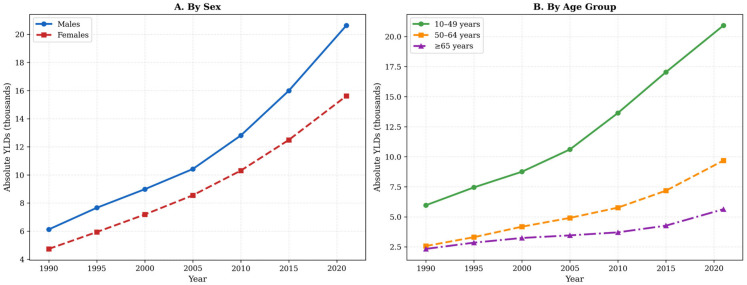
Trends in absolute stroke YLDs in Saudi Arabia, 1990–2021. Panel (**A**): By sex, with separate lines for males (blue, solid circles) and females (red, dashed squares). Panel (**B**): By age group (10–49 years, 50–64 years, and ≥65 years). Absolute YLD counts were summed across available age groups (≥10 years). GBD 2021 estimates.

Despite declining or stable age-standardized rates, absolute stroke-related YLDs increased more than threefold, from approximately 10,900 (95% UI: 8100–13,900) in 1990 to 36,245 (95% UI: 26,600–46,100) in 2021 (Table 1; Figure 2). This divergence between standardized rates and absolute counts reflects concurrent population growth and demographic aging during the study period.

### 3.2. Disparities in Stroke Burden by Subtype, Sex, and Age, 2021

In 2021, ischemic stroke was the predominant contributor to disability across all age groups, with an age-standardized YLD rate of 101.4 per 100,000 (95% UI: 74.8–128.9), accounting for 78.0% of total stroke YLDs (Table 2). Intracerebral hemorrhage contributed 20.2 per 100,000 (15.6%), and subarachnoid hemorrhage 8.3 per 100,000 (6.4%).

YLD rates increased progressively with age for all subtypes and both sexes. For ischemic stroke, males had higher age-standardized YLD rates than females (105.5 versus 95.5 per 100,000), with the largest absolute sex difference observed among individuals aged 75 years or older (males: 842.4 per 100,000; females: 492.7 per 100,000). For total stroke, the age-standardized YLD rate was slightly higher in males than in females (131.4 versus 128.0 per 100,000).

A distinct pattern emerged for hemorrhagic stroke in younger adults. Among individuals aged 20–49 years, females had higher YLD rates than males for intracerebral hemorrhage, with female-to-male rate ratios ranging from 1.17 to 1.24. The disparity was more pronounced for subarachnoid hemorrhage, with female-to-male rate ratios ranging from 1.51 in those aged 20–24 years to 1.68 in those aged 45–49 years. These ratios are unadjusted and should be interpreted cautiously, as the male population denominator is expanded by a predominantly male expatriate workforce. Among adults aged 65 years or older, the female excess in subarachnoid hemorrhage persisted; in those aged 75 years or older, YLD rates were 34.3 per 100,000 in females compared with 13.3 per 100,000 in males.

**Figure 3:**
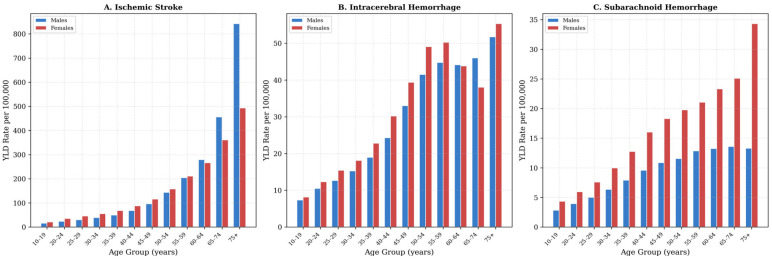
Stroke YLD rates per 100,000 by subtype and age group, stratified by sex in Saudi Arabia, 2021. Panel (**A**): Ischemic stroke. Panel (**B**): Intracerebral hemorrhage. Panel (**C**): Subarachnoid hemorrhage. Blue bars represent males; red bars represent females. GBD 2021 estimates.

Absolute YLD counts in 2021 totaled 36,245 across individuals aged 10 years or older (Table 3). Males accounted for 20,630 YLDs (56.9%) and females for 15,615 (43.1%). Among males, ischemic stroke comprised 73.2% of total YLDs (15,106 of 20,630), followed by intracerebral hemorrhage (19.8%; 4077) and subarachnoid hemorrhage (7.0%; 1446). Among females, ischemic stroke accounted for 70.2% of YLDs (10,962 of 15,615), intracerebral hemorrhage for 19.8% (3094), and subarachnoid hemorrhage for 10.0% (1559). The higher proportional contribution of subarachnoid hemorrhage among females is consistent with the sex-specific rate patterns described above (Figure 3).

### 3.3. Projected Stroke-Related Disability and Rehabilitation Needs, 2022–2030

**Figure 4:**
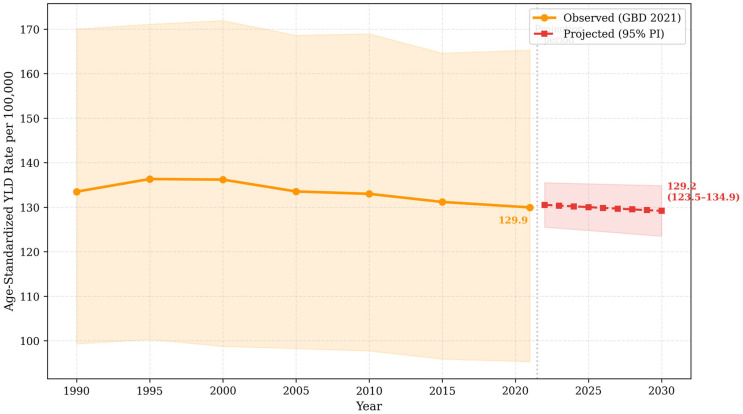
Projected age-standardized stroke YLD rates in Saudi Arabia, 2021–2030. Orange circles with solid line: Observed GBD 2021 estimates (1990–2021). Red squares with dashed line: Linear projections (2022–2030). Shaded areas represent 95% uncertainty intervals (observed) and 95% prediction intervals (projected). A vertical dotted line indicates the transition from observed to projected data.

The age-standardized stroke YLD rate is projected to remain stable through 2030, declining slightly from 129.9 per 100,000 in 2021 to 129.2 per 100,000 (95% prediction interval [PI]: 123.5–134.9) (Figure 4). In contrast, absolute stroke-related YLDs are projected to increase from 36,245 in 2021 to approximately 40,764 (95% PI: 33,808–47,720) in 2030, reflecting continued population growth and aging.

**Figure 5:**
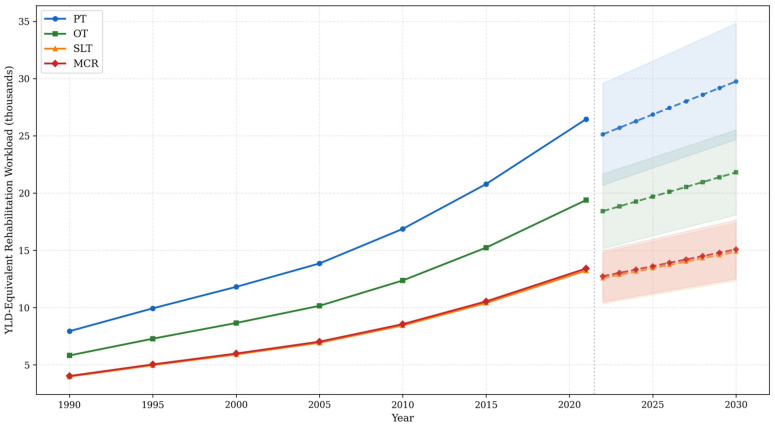
Projected stroke rehabilitation needs by rehabilitation modality in Saudi Arabia, 1990–2030. PT = physiotherapy (blue); OT = occupational therapy (green); SLT = speech–language therapy (yellow); MCR = multidisciplinary comprehensive rehabilitation (red). Solid lines: observed (1990–2021). Dashed lines: projected (2022–2030). Shaded areas: 95% prediction intervals. Units: YLD-equivalent rehabilitation workload (thousands).

Under base-case assumptions regarding the distribution of disability severity and service utilization (30% mild, 50% moderate, 20% severe), the estimated YLD-equivalent rehabilitation workload in 2021 was 26,459 for PT, 19,391 for OT, 13,229 for speech–language therapy, and 13,411 for multidisciplinary comprehensive rehabilitation. By 2030, projected demand increases to 29,758 (95% PI: 24,680–34,836) for PT, 21,809 (95% PI: 18,087–25,530) for OT, 14,879 (95% PI: 12,340–17,418) for SLT, and 15,083 (95% PI: 12,509–17,656) for multidisciplinary rehabilitation (Figure 5). This corresponds to a 12.5% increase in projected demand for both physiotherapy and multidisciplinary rehabilitation between 2021 and 2030.

**Figure 6:**
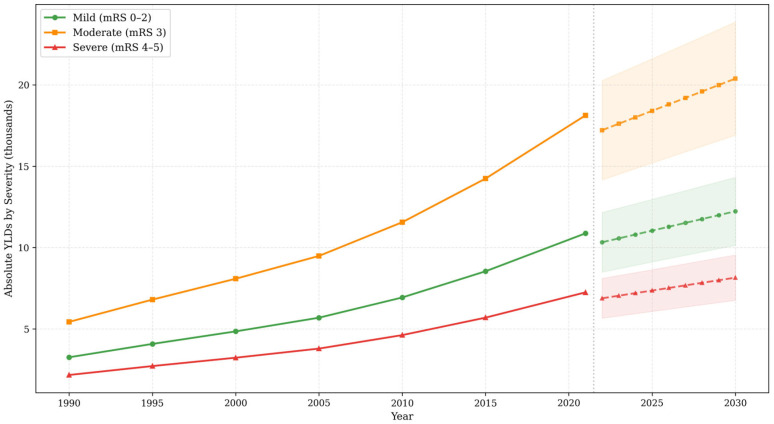
Projected stroke rehabilitation needs by severity level in Saudi Arabia, 1990–2030. Green: Mild (mRS 0–2). Orange: Moderate (mRS 3). Red: Severe (mRS 4–5). Solid lines: Observed. Dashed lines: Projected. Shaded areas: 95% prediction intervals. Units: Absolute YLDs by severity category (thousands).

When stratified by disability severity, moderate disability (modified Rankin Scale score 3) accounted for the largest proportion of projected YLDs across all years, by definition representing 50% of total YLDs (Figure 6). By 2030, projected YLDs are estimated at 20,382 (95% PI: 16,904–23,860) in the moderate category, 12,229 in the mild category, and 8153 in the severe category.

The projected increase in absolute rehabilitation demand, despite stable age-standardized YLD rates, reflects demographic expansion and population aging. Because age standardization adjusts for changes in age structure, it does not capture the service demand generated by an aging population. These projections assume continuation of recent age-standardized trends and may therefore represent conservative estimates.

### 3.4. Sensitivity Analysis

Rehabilitation demand estimates were sensitive to assumptions regarding disability severity and service utilization (Supplementary Table S3). Under Scenario A, which applied a more severe distribution for hemorrhagic stroke (20% mild, 40% moderate, 40% severe), projected demand increased by 6.8% for multidisciplinary rehabilitation, 5.0% for SLT, 3.9% for OT, and 1.9% for PT relative to the base case. The modest magnitude of change reflects the fact that hemorrhagic stroke accounted for 28.1% of total YLDs.

Under Scenario B, which varied utilization probabilities by ±15%, total rehabilitation demand changed proportionally. At the upper bound, projected demand reached 30,428 for PT, 22,300 for OT, 15,214 for SLT, and 15,422 for multidisciplinary rehabilitation. At the lower bound, corresponding values were 22,490, 16,482, 11,245, and 11,399, respectively. Across all scenarios, PT remained the modality with the highest projected demand, and moderate disability remained the largest severity category.

## 4. Discussion

This study provides a comprehensive assessment of stroke burden and projected rehabilitation demand in Saudi Arabia using GBD 2021 data, extending previous work by incorporating YLLs, disaggregated estimates of SAH, and a structured mapping of rehabilitation needs [1,2,3,4,24]. Three principal findings emerged.

First, a marked divergence between mortality and disability was observed. Between 1990 and 2021, the age-standardized YLL rate declined by 41.0%, from 3209.0 to 1893.4 per 100,000. In contrast, the age-standardized YLD rate declined only modestly by 2.7%, from 133.5 to 129.9 per 100,000. Absolute YLDs more than tripled during this period, increasing from approximately 10,900 to 36,245, largely driven by population growth and demographic aging.

Second, substantial disparities by subtype, sex, and age were evident in 2021. Ischemic stroke accounted for 78.0% of stroke-related YLDs. In contrast, SAH demonstrated a pronounced female-to-male YLD rate ratio of 1.5–1.7 among individuals aged 15–49 years, exceeding the corresponding intracerebral hemorrhage (ICH) ratio of 1.1–1.2.

Third, projections to 2030 indicate that physiotherapy (PT; 29,758 YLD-equivalent units) and multidisciplinary comprehensive rehabilitation (MCR; 15,083 YLD-equivalent units) will constitute the largest areas of projected need, underscoring the requirement for strategic expansion of rehabilitation capacity.

These findings depart from the framing in earlier national analyses: Basri et al. [7] and Mahfouz et al. [8] documented rising stroke incidence and mortality; the present data show a 41.0% decline in age-standardized YLLs, consistent with expanded access to thrombolysis, thrombectomy, and dedicated stroke units over the study period [9,13,14]. YLD rates, however, fell by only 2.7% over the same interval, and age-standardized ischemic stroke YLD rates showed no meaningful change at all. Gains in acute survival have not been matched by equivalent reductions in long-term disability, and an increasing number of patients now live with persistent functional impairment following stroke, shifting the residual burden from acute mortality toward chronic disability.

Disaggregation by stroke subtype reveals that the modest overall decline in YLD rates conceals divergent trends. The age-standardized ischemic stroke YLD rate remained essentially unchanged between 1990 and 2021, increasing slightly from 98.9 to 101.4 per 100,000 (+2.5%; average annual percentage change [AAPC] +0.05%, *p* = 0.323). In contrast, YLD rates declined by 19.8% for ICH and by 11.1% for SAH during the same period. Because ischemic stroke accounted for 78.0% of all stroke-related YLDs, the modest overall decline was driven primarily by reductions in hemorrhagic stroke. The dominant ischemic subtype showed no meaningful improvement in disability burden. This subtype-specific stagnation has not been emphasized in prior Saudi or regional analyses [7,8,27] and has direct implications for rehabilitation planning. The principal population requiring rehabilitation, ischemic stroke survivors, has not experienced reductions in disability despite declining incidence (−21.4%).

Stroke burden increased substantially with advancing age across all subtypes. Among males aged ≥ 70 years, ischemic stroke YLD rates reached 842.5 per 100,000, compared with 65.0 per 100,000 for hemorrhagic stroke subtypes combined [1,2,4]. Disaggregation of hemorrhagic stroke into ICH and SAH revealed distinct sex-specific patterns among younger adults. In individuals aged 15–49 years, the female-to-male YLD rate ratio for SAH ranged from 1.5 to 1.7, substantially exceeding the corresponding ICH ratio of 1.1–1.2. This pattern aligns with evidence of higher intracranial aneurysm prevalence and increased rupture risk among premenopausal women [28,29]. Demographic factors in Saudi Arabia, including the large influx of young male expatriate workers, may further accentuate these observed ratios. In contrast, the more modest female excess in ICH within this age group is largely associated with hypertensive disorders of pregnancy, eclampsia, and peripartum coagulopathy [30]. Earlier literature attributed the excess of hemorrhagic stroke among females primarily to pregnancy-related hypertension [2,4,31]. The present disaggregated findings indicate that SAH and ICH require distinct preventive strategies.

Comparison with neighboring Gulf Cooperation Council (GCC) countries shows both shared patterns and country-specific differences. Studies from the United Arab Emirates, Kuwait, and Qatar report that ischemic stroke accounts for the majority of cases and that hemorrhagic stroke disproportionately affects younger adults [8,27]. Absolute YLDs in Saudi Arabia are higher, reflecting its larger population and more advanced stage of demographic transition. Regional comparisons require methodological caution: earlier GCC analyses, including Jaberinezhad et al. [27], used GBD 2019 estimates with different modeling inputs and covariate specifications for Saudi Arabia. These differences between GBD cycles reflect revisions to underlying data sources, population envelopes, and cause-of-death modeling. Comparison of absolute values across GBD iterations is therefore inappropriate; this analysis compares directional trends and patterns rather than point estimates, which is the methodologically appropriate approach when estimates derive from different GBD cycles.

The mortality–disability divergence observed here is consistent with global GBD analyses showing declining stroke mortality and incidence alongside growth in stroke survivor populations and associated disability [1,2,3,4,32,33,34]. Similar trends have been reported in high- and middle-income countries where advances in acute stroke management have reduced case fatality but increased the prevalence of chronic post-stroke impairment [3,4,32]. The magnitude of divergence in Saudi Arabia (−41.0% YLLs versus −2.7% YLDs) exceeds global averages and likely reflects the rapid expansion of acute stroke care infrastructure over the past two decades, including the establishment of dedicated stroke units and increased thrombolysis and thrombectomy capacity [9,13,14]. The persistence of ischemic stroke–related disability, which this analysis documents for the first time in the national literature, suggests that equivalent advances in secondary prevention or post-acute rehabilitation have not accompanied gains in acute care. Both require considerable strengthening, and neither has been systematically evaluated against the scale of need quantified here [5,6,25,26].

Provincial studies in Aseer, Taif, and other regions of Saudi Arabia document high first-stroke incidence and a heavy burden of vascular risk factors, including hypertension, diabetes mellitus, dyslipidemia, and obesity [35,36]. A study in the Aseer region reported an age-adjusted first-ever stroke incidence rate of 29.8 per 100,000 [35]. Alhazzani et al. [31] identified hypertension and diabetes mellitus as the primary modifiable risk factors. The present data place these provincial findings in a national context: acute survival has improved, but disability has increased correspondingly, and the discrepancy between reductions in stroke incidence and in disability indicates that rehabilitation services have not expanded in proportion to need. By integrating GBD 2021 severity estimates with rehabilitation modality mapping, this analysis advances prior work by quantifying demand in YLD-equivalent units across therapeutic approaches, rather than relying solely on incidence counts [5,6,25,26].

The disproportionate burden of hemorrhagic stroke among younger females warrants targeted preventive strategies. The elevated female-to-male YLD ratio for SAH (1.5–1.7) in individuals aged 15–49 years reflects a well-established epidemiological pattern [28,29]. Systematic reviews consistently report higher incidence and prevalence of intracranial aneurysms in women, with increased rupture risk during premenopausal years. These findings support consideration of targeted neurovascular screening among women with established risk factors, including a family history of aneurysmal SAH, autosomal dominant polycystic kidney disease, or connective tissue disorders. Concurrent management of modifiable risk factors, particularly smoking cessation and blood pressure control, remains essential [28]. For ICH, prevention should focus on optimal management of pregnancy-related hypertension through preeclampsia screening, antihypertensive therapy, and integrated obstetric–neurological care pathways [30]. These subtype-specific strategies refine earlier undifferentiated approaches to hemorrhagic stroke prevention among young females [2,4,31].

### 4.1. Implications for Health System Planning and Vision 2030

Saudi Arabia’s Vision 2030 and the Health Sector Transformation Program (HSTP) prioritize noncommunicable disease prevention, integrated care models, value-based delivery, and expansion of community-based rehabilitation services [15,18,37]. These policy frameworks provide the planning context within which the present projections should be interpreted. The 2030 estimates (PT: 29,758 and MCR: 15,083 YLD-equivalent units) are conservative lower-bound figures, as projections derived from declining age-standardized rates will understate absolute demand in a rapidly aging population. The proportion of Saudi residents aged ≥65 years is projected to approximately double between 2021 and 2030 [24], and older adults experience both higher stroke incidence and greater disability severity per event; actual rehabilitation demand will therefore likely exceed these projections, and planners should treat the 2030 figures as a minimum rather than a target.

These findings are consistent with the World Stroke Organization Global Stroke Action Plan 2018–2030 [38] and the European Stroke Organisation Action Plan for Stroke in Europe 2018–2030 [39], both of which identify demographic aging as a force capable of offsetting primary prevention gains, sustaining or increasing absolute stroke burden despite declining age-standardized rates. The Saudi data illustrate this pattern at the national level: a 21.4% decline in age-standardized stroke incidence and a 41.0% decline in age-standardized YLLs were accompanied by a tripling of absolute YLDs between 1990 and 2021. As a high-income country in rapid demographic transition, Saudi Arabia may serve as an informative reference for other GCC and Middle East and North Africa countries where similar demographic changes are anticipated.

The economic consequences follow directly from the disability data. Per-patient stroke costs include direct expenditures—acute care, rehabilitation, medications, and outpatient follow-up—and indirect costs—productivity loss and informal caregiving [40]. Rising YLDs combined with high per-patient costs indicate a growing cumulative economic burden as the population ages. Community-based rehabilitation, outpatient multidisciplinary programs, and telerehabilitation offer more cost-effective alternatives to prolonged inpatient care [17,41] and should be prioritized within the HSTP. Saudi Stroke Standards have identified gaps in community-based rehabilitation and early supported discharge [14,19]; the present findings quantify the scale of unmet need associated with those deficiencies.

Growing post-stroke disability carries workforce implications as well. The projected dominance of PT demand (29,758 units) over MCR demand (15,083 units) reflects the higher prevalence of mild-to-moderate disability in the ischemic stroke population; however, MCR projections are sensitive to severity assumptions and are likely underestimated for hemorrhagic subtypes. Rising overall rehabilitation demand, particularly for moderate-to-severe disability, will require expanding the workforce, including physiotherapists, occupational therapists, and speech-language therapists who deliver multidisciplinary care [10,16,26]. The YLD-equivalent workloads reported here serve as demand proxies. Translating them into operational workforce planning requires comparison with current supply metrics, the number of physiotherapists registered with the Saudi Commission for Health Specialties, and the stroke rehabilitation sessions recorded by the Ministry of Health and other public and private providers [5,6,10,11]. Incorporating disability-informed, subtype-specific, and sex-stratified rehabilitation indicators within Vision 2030 and HSTP monitoring frameworks would enable systematic evaluation of service capacity, quality, and outcomes over time [15,18,41].

### 4.2. Clinical and Public Health Implications

Persistent stroke-related disability despite declining mortality indicates a clear need for long-term rehabilitation planning. The stagnation of ischemic stroke YLD rates (+2.5%) shows that secondary prevention and rehabilitation for ischemic stroke survivors—the largest rehabilitation population—are insufficient. Rehabilitation programs must address motor, cognitive, communication, emotional, and vocational domains, with particular attention to younger adults affected by hemorrhagic stroke subtypes [25,26]. Programs should ensure early initiation, adequate treatment intensity, and sustained follow-up to support functional recovery and community reintegration. Evidence-based interventions, including early supported discharge, intensified pharmacological secondary prevention, and structured cognitive and psychosocial rehabilitation, require broader implementation [14,19,25].

From a public health perspective, control of hypertension, diabetes mellitus, dyslipidemia, and obesity remains central to stroke prevention, alongside public education to improve stroke recognition and timely access to thrombolysis and thrombectomy [2,4,13,31]. Integrating stroke prevention clinics and cardiovascular risk assessment into primary care, aligned with Vision 2030 noncommunicable disease targets and Health Cluster reforms, may reduce future incidence and disability burden. The convergence of hypertension, diabetes mellitus, and obesity as dominant risk factors in Saudi Arabia provides a rationale for unified vascular risk programs rather than stroke-specific interventions alone [13,14,15,41].

Among younger females, the disaggregated hemorrhagic stroke data support two prevention pathways: (1) neurovascular screening and aneurysm management for prevention of SAH, particularly in women with a family history of aneurysmal SAH, autosomal dominant polycystic kidney disease, or connective tissue disorders, and in those with additional modifiable risk factors such as smoking and uncontrolled hypertension. Translating this evidence into clinical practice requires dedicated screening studies, as the cost-effectiveness of screening programs in the Saudi population has not been established [28,29]; and (2) enhanced management of pregnancy-related hypertension for prevention of ICH, including preeclampsia screening, antihypertensive treatment during pregnancy, and postpartum vascular monitoring [30]. Programs addressing hypertensive disorders of pregnancy, contraceptive counseling, and postpartum follow-up may reduce preventable hemorrhagic events. These findings support parallel implementation of stroke prevention and rehabilitation strategies addressing both upstream risk determinants and the downstream consequences of stroke [5,6,34,42].

### 4.3. Strengths and Limitations

This is, to our knowledge, the first published national analysis using GBD 2021 estimates to project stroke-related rehabilitation demand in Saudi Arabia stratified by modality and severity. The analysis disaggregates hemorrhagic stroke into ICH and SAH, stratifies outcomes by sex and age, and evaluates both YLL and YLD trends to characterize mortality–disability divergence. Severity-based rehabilitation modality mapping with 95% prediction intervals and sensitivity analyses—including a hemorrhagic-weighted severity distribution and ±15% variation in utilization probability—provides a transparent and reproducible framework for strategic planning.

Several limitations constrain interpretation. First, GBD 2021 estimates are model-based and derived from heterogeneous sources; uncertainty intervals reflect residual model uncertainty rather than observed variability [1,2,3,4,43]. Second, GBD data for Saudi Arabia cover seven time points (1990, 1995, 2000, 2005, 2010, 2015, 2021) rather than continuous annual observations. Log-linear models fitted to these data have limited degrees of freedom (df = 5), yielding wider confidence intervals and reduced capacity to detect non-linear trends or inflection points, such as an acceleration in burden associated with the rapid demographic aging expected after 2021. Third, projections based on declining age-standardized rates will understate absolute demand in a rapidly aging population; the proportion of residents aged ≥65 years is projected to double between 2021 and 2030, and these estimates should be treated as conservative lower bounds.

Fourth, the base-case severity distribution (30% mild, 50% moderate, 20% severe) was applied uniformly across all subtypes and may understate disability associated with hemorrhagic stroke. Sensitivity analysis using a hemorrhagic-weighted distribution (20% mild, 40% moderate, 40% severe for ICH and SAH) produced a 7.0% increase in MCR demand, indicating sensitivity to severity assumptions while maintaining consistent directional findings (Supplementary Table S3). Fifth, utilization probabilities were derived from international rehabilitation guidelines and regional practice surveys [17,25,26] rather than from Saudi-specific data; utilization may differ due to variation in service availability, workforce capacity, referral practices, and cultural factors. Validation using national stroke registry data is required.

Sixth, YLD-equivalent workloads are planning proxies and do not directly correspond to therapy sessions, full-time equivalents of the workforce, or facility capacity [5,6]; they estimate the relative scale and trajectory of demand rather than operational requirements. Seventh, GBD estimates for Saudi Arabia include the entire resident population, approximately 38–40% of whom are non-national expatriates. This group, predominantly young adult males, compresses age-standardized rates in younger cohorts, potentially distorting female-to-male ratios. Because expatriate workers frequently use private insurance or are repatriated for severe disability management, total population estimates may overstate the rehabilitation workload for public-sector Ministry of Health facilities, and the estimates presented here should be interpreted as national population-level figures rather than projections of Ministry of Health demand specifically.

Despite these limitations, the study provides a standardized framework for estimating stroke rehabilitation needs in Saudi Arabia. The extended time horizon, consistent methodology, integration of both mortality and disability measures, and explicit alignment with Vision 2030 planning priorities contribute to evidence-informed health system planning and capacity development [15,18,41].

### 4.4. Future Research

Several priorities for future research emerge. First, scenario-based projection models should incorporate demographic changes (population aging and urbanization), trends in risk factors (including rising obesity and diabetes prevalence), health system performance indicators (thrombolysis and thrombectomy rates, and rehabilitation coverage), and policy interventions, such as the expansion of community rehabilitation under Vision 2030. These approaches could generate alternative scenarios for rehabilitation investment planning beyond single-trend extrapolation.

Second, empirical validation is needed by linking national stroke registry data with rehabilitation service records. Integration of stroke incidence, rehabilitation utilization, and functional outcomes would permit calibration of modeling assumptions and estimation of the gap between projected need and actual service delivery [14,19,20].

Third, economic analyses quantifying direct and indirect costs of stroke-related disability in Saudi Arabia, including health care expenditures, productivity losses, and informal caregiving, would strengthen the economic rationale for rehabilitation investment and support cost-effectiveness comparisons across delivery models, including inpatient, community-based, and telerehabilitation approaches [40,41].

Fourth, qualitative and mixed-methods studies examining barriers to rehabilitation access, using predictive modeling and technology, patient and caregiver preferences, and workforce readiness would inform implementation strategies for scaling rehabilitation services within the evolving Health Cluster framework [14,16,17,20]. These investigations would complement population-level estimates with contextual evidence relevant to service design and delivery.

## 5. Conclusions

Between 1990 and 2021, the age-standardized rate of years of life lost due to stroke in Saudi Arabia declined by 41.0%, reflecting improvements in acute survival. The age-standardized rate of years lived with disability fell by only 2.7% over the same period, and the absolute number of stroke-related YLDs more than tripled, from approximately 10,900 (95% UI: 8100–13,900) to 36,245 (95% UI: 26,600–46,100), driven by population growth and demographic aging. Acute care gains reduced premature mortality while the population living with chronic post-stroke disability grew substantially.

Disability patterns differed by subtype. Age-standardized YLD rates fell for hemorrhagic stroke: by 19.8% for intracerebral hemorrhage and by 11.1% for subarachnoid hemorrhage. Ischemic stroke, which accounted for 78.0% of all stroke-related YLDs, showed no change in disability rates over three decades. This stagnation identifies ischemic stroke survivors as the population for whom secondary prevention and post-acute rehabilitation have been least effective and where capacity investment is most needed.

Among adults aged 15–49 years, the female-to-male YLD rate ratio for subarachnoid hemorrhage was 1.5–1.7, exceeding the corresponding ratio for intracerebral hemorrhage, which was 1.1–1.2. This difference reflects distinct pathophysiological profiles of intracranial aneurysm biology versus pregnancy-related hypertension and argues against managing the two subtypes under a single hemorrhagic stroke prevention framework. Rate estimates in working-age cohorts should be interpreted with caution, given the male-predominant expatriate workforce in Saudi Arabia, which compresses male denominators in this age group.

Projections to 2030 place physiotherapy and multidisciplinary comprehensive rehabilitation as the two highest-demand service categories by YLD-equivalent workload. These figures are estimates of relative demand, not workforce or infrastructure targets. Translating them into operational plans requires data on current rehabilitation service capacity that were not available for this analysis.

Declining stroke mortality and growing disability burden are not contradictory trends; they are the predictable result of effective acute care in an aging population without proportionate investment in rehabilitation. The data presented here quantify that gap by subtype, sex, and age. Whether existing services are adequate to meet it is a question that requires linking national stroke registries with rehabilitation utilisation records, a priority for future research.

## Figures and Tables

**Table 1:** Age-standardized stroke metrics in Saudi Arabia, 1990 vs. 2021 (both sexes).

Measure	1990 Rate (95% UI)	2021 Rate (95% UI)	% Change	AAPC % (95% CI)	*p*-Value
Incidence	166.3 (152.0–182.3)	130.7 (119.4–141.7)	−21.4%	−0.86 (−1.28, −0.44)	0.004
Prevalence	982.4 (933.0–1031.9)	965.2 (924.9–1006.4)	−1.8%	−0.10 (−0.20, +0.00)	0.056
YLLs	3209.0 (2507.5–4006.7)	1893.4 (1556.8–2314.4)	−41.0%	−1.76 (−2.07, −1.45)	<0.001
YLDs	133.5 (99.3–170.0)	129.9 (95.3–165.3)	−2.7%	−0.13 (−0.24, −0.02)	0.032
DALYs	3342.4 (2659.9–4148.0)	2023.3 (1687.1–2452.5)	−39.5%	−1.67 (−1.97, −1.38)	<0.001

Abbreviations: AAPC, average annual percentage change; CI, confidence interval; DALYs, disability-adjusted life years; UI, uncertainty interval; YLDs, years lived with disability; YLLs, years of life lost. Rates are per 100,000 population. All rates are age-standardized to the GBD reference population. Log-linear regression on 7 milestone-year observations (1990–2021; df = 5).

**Table 2:** Age-specific stroke YLD rates per 100,000 by subtype, sex, and age group in Saudi Arabia, 2021.

Subtype	Sex	10–19 Years	20–24 Years	25–29 Years	30–34 Years	35–39 Years	40–44 Years	45–49 Years	65–74 Years	75+ Years
Ischemic stroke										
	Male	15.4	23.5	29.9	39.0	49.7	68.2	96.1	456.2	842.5
	Female	21.2	35.0	45.6	55.2	68.1	88.0	115.5	360.8	492.7
Intracerebral hemorrhage (ICH)										
	Male	7.3	10.5	12.7	15.3	18.9	24.3	33.0	46.0	51.7
	Female	8.2	12.3	15.4	18.1	22.8	30.2	39.4	38.1	55.3
Subarachnoid hemorrhage (SAH)										
	Male	2.8	3.9	5.0	6.3	7.9	9.6	10.9	13.6	13.3
	Female	4.3	5.9	7.6	10.0	12.7	16.0	18.3	25.1	34.3
Total stroke										
	Male	25.6	38.0	47.6	60.6	76.6	102.0	140.0	515.8	907.5
	Female	33.7	53.3	68.6	83.3	103.6	134.1	173.1	423.9	582.3

Note: YLD rates per 100,000 population. Selected age groups shown; intermediate age groups (50–54, 55–59, 60–64 years) are available in the Supplementary Data (Supplementary Table S4). Estimates from the GBD 2021 study.

**Table 3:** Estimated absolute YLDs by stroke subtype, sex, and age group in Saudi Arabia, 2021.

Subtype	Sex	10–19 Years	20–24 Years	25–29 Years	30–34 Years	35–39 Years	40–44 Years	45–49 Years	65–74 Years	75+ Years	Total
Ischemic stroke											
	Male	429	420	661	1043	1391	1679	1723	2200	1235	15,106
	Female	490	474	733	989	1180	1260	1257	1071	474	10,962
Intracerebral hemorrhage (ICH)											
	Male	204	188	280	408	530	598	592	222	76	4077
	Female	189	167	248	324	395	432	428	113	53	3094
Subarachnoid hemorrhage (SAH)											
	Male	79	70	111	170	221	236	195	66	20	1446
	Female	100	80	122	179	221	229	199	75	33	1560
Total stroke											
	Male	712	678	1052	1620	2142	2513	2510	2487	1330	20,630
	Female	779	721	1103	1492	1796	1922	1885	1259	560	15,615

Note: Absolute YLD counts are rounded to the nearest integer. Totals include all available age groups (≥10 years), including intermediate groups (50–54, 55–59, 60–64 years), which are detailed in Supplementary Table S4. Male + Female totals = 36,245 (95% UI: 26,600–46,100) for total stroke. GBD 2021 estimates.

## Data Availability

GBD 2021 summary estimates are publicly available from the Institute for Health Metrics and Evaluation (IHME) GBD results tool: https://vizhub.healthdata.org/gbd-results/ (accessed on 14 March 2026). National population data and projections are available from the General Authority for Statistics, Saudi Arabia (https://www.stats.gov.sa/) (accessed on 14 March 2026).

## Supplementary Materials

The following supporting information can be downloaded at https://www.mdpi.com/article/10.3390/jcm15062382/s1. Table S1: GATHER checklist; Table S2: STROBE checklist; Table S3: Sensitivity analysis using two alternative scenarios; Table S4: Detailed GBD 2021 stroke estimates (including Age-Standardized Rate Trends 1990–2021, Absolute YLDs 2021, and Age-Specific YLD Rates for intermediate age groups); Table S5: Age-Standardized Rates and Average Annual Percentage Change (AAPC) by Stroke Subtype, All Measures—Saudi Arabia, 1990 vs. 2021.

## Abbreviations

The following abbreviations are used in this manuscript:

AAPC	Average annual percentage change
DALY	Disability-adjusted life year
GASTAT	General Authority for Statistics (Saudi Arabia)
GATHER	Guidelines for Accurate and Transparent Health Estimates Reporting (Methods)
GBD	Global Burden of Disease
GBD 2021	Global Burden of Disease Study 2021
GCC	Gulf Cooperation Council (Discussion)
HSTP	Health Sector Transformation Program
ICD	International Classification of Diseases
ICH	Intracerebral hemorrhage
IHME	Institute for Health Metrics and Evaluation
MCR	Multidisciplinary comprehensive rehabilitation
MENA	Middle East and North Africa (Discussion)
mRS	Modified Rankin Scale
NCD	Noncommunicable disease (Discussion)
OT	Occupational therapy
PI	Prediction interval (Results)
PT	Physiotherapy
SA	Saudi Arabia
SAH	Subarachnoid hemorrhage
SLT	speech–language therapy
STROBE	Strengthening the Reporting of Observational Studies in Epidemiology
UIs	Uncertainty intervals
WHO	World Health Organization
YLDs	Years lived with disability
YLLs	Years of life lost (used in every section)
