# Supplementary material for: Trends in Stroke Burden and Rehabilitation Demand in Saudi Arabia, 1990–2021, with Projections to 2030: A National Analysis Using GBD 2021 Data
Source: J Clin Med. 2026 Mar 20;15(6):2382. doi: 10.3390/jcm15062382 (PMC13026204; doi:10.3390/jcm15062382)
Supplement: Supplementary file 1 [file jcm-15-02382-s001.zip › jcm-4171092-supplementary.pdf]

The five supplementary files listed in the manuscript:

1. **Table S1**: STROBE checklist.
2. **Table S2**: GATHER checklist.
3. **Table S3**: Sensitivity analysis using two alternative scenarios.
4. **Table S4**: Detailed GBD 2021 stroke estimates (including Age-Standardized Rate Trends 1990–2021, Absolute YLDs 2021, and Age-Specific YLD Rates for intermediate age groups).
5. **Table S5**: Age-Standardized Rates and Average Annual Percentage Change (AAPC) by Stroke Subtype, All Measures: Saudi Arabia, 1990 vs. 2021

Supplementary Table S1: The STROBE checklist

Section	Item No.	Recommendation	Page No(s). & Manuscript Location
**Title and Abstract**			
Title and Abstract	1a	Indicate the study's design with a commonly used term in the title or abstract	1 (Title / Abstract)
Title and Abstract	1b	Provide an informative and balanced summary of what was done and what was found	1 (Abstract)
**Introduction**			
Background/Rationale	2	Explain the scientific background and rationale for the investigation	2–3 (Section 1. Introduction)
Objectives	3	State-specific objectives, including any prespecified hypotheses	3 (Section 1. Introduction)
**Methods**			
Study Design	4	Present key elements of the study design early in the paper	3 (Section 2.1. Study Design)
Setting	5	Describe the setting, locations, and relevant dates, including data collection periods	3–4 (Section 2.2. Data Sources)
Participants	6a	Describe eligibility criteria and sources/methods of participant selection (GBD population framework)	3–4 (Section 2.2.3. Population Data)
Participants	6b	Matching criteria (if applicable)	NA
Variables	7	Define outcomes, predictors, confounders, and diagnostic criteria	4–5 (Sections 2.2.2 & 2.3. Measures)
Data Sources/ Measurement	8*	Describe data sources and measurement methods; comparability across groups	3–6 (Section 2.2. Data Sources)
Bias	9	Describe efforts to address potential sources of bias	15 (Section 4.3. Strengths and Limitations)
Study Size	10	Explain how the study size was determined	3–4 (Section 2.2.3. Population Data)
Quantitative Variables	11	Explain the handling of quantitative variables and groupings	4–6 (Sections 2.3 & 2.7.1. Severity Distribution)
Statistical Methods	12a	Describe statistical methods and adjustments	4–6 (Sections 2.4. Trend Analysis & 2.6)
Statistical Methods	12b	Describe methods for subgroup and interaction analyses	5–6 (Section 2.5. Disparity Analysis)
Statistical Methods	12c	Explain handling of missing data (GBD modeling framework)	4–6 (Section 2.2.1. GBD 2021 Estimates)
Statistical Methods	12d	Loss to follow-up (if applicable)	NA
Statistical Methods	12e	Describe sensitivity analyses	15 (Sections 2.7.1 & Sensitivity Analysis)
**Results**			
Participants	13a	Report numbers at each stage of the study	NA (population-level data)
Participants	13b	Reasons for non-participation	NA
Participants	13c	Flow diagram	NA
Descriptive Data	14a	Report population characteristics and key variables	Tables 1–3 (Sections 3.1 & 3.2)

Descriptive Data	14b	Report missing data for variables	NA (modeled estimates)
Descriptive Data	14c	Follow-up time (if applicable)	NA
Outcome Data	15	Report outcome measures or summary estimates	Tables 1–3; Figures (Sections 3.1 & 3.2)
Main Results	16a	Provide estimates with precision (e.g., 95% uncertainty intervals)	Tables 1–3; Figures (Sections 3.1 & 3.2)
Main Results	16b	Report category boundaries for grouped variables	4–5 (Sections 2.3 & 2.7)
Main Results	16c	Translate relative into absolute risk, if relevant	NA
Other Analyses	17	Report subgroup and sensitivity analyses	5–6; 15 (Sensitivity Analysis)
**Discussion**			
Key Results	18	Summarize key results in relation to objectives	14 (Section 4. Discussion)
Limitations	19	Discuss study limitations and potential bias	15 (Section 4.3. Strengths and Limitations)
Interpretation	20	Provide a cautious overall interpretation considering the evidence	14–15 (Section 4. Discussion)
Generalisability	21	Discuss the external validity of findings	15 (Sections 4.1 & 4.2. Implications)
**Other Information**			
Funding	22	Report funding sources and the role of funders	16 (Funding section)

Supplementary Table S2: The Guidelines for Accurate and Transparent Health Estimates Reporting (GATHER) checklist

Item No.	Checklist Item	Page # & Exact Manuscript Location
**Objectives and funding**		
1	Define indicators, populations (age, sex, geography), and the time period of estimates	3–6 (Section 2.2.1. GBD 2021 Stroke Estimates; Section 2.3. Measures and Metrics)
2	List funding sources	16 (Funding)
**Data inputs (synthesized data)**		
3	Describe how the data were identified and accessed	3 (Section 2.2.1. GBD 2021 Stroke Estimates; Data Availability Statement)
4	Specify inclusion/exclusion criteria and ad-hoc exclusions	3–4 (Section 2.2.2. Case Definition and Coding)
5	Describe included data sources and key characteristics (population, methods, years, age/sex, case definition)	3–6 (Section 2.2. Data Sources)
6	Identify data sources with potential bias	15 (Section 4.3. Strengths and Limitations)
**Other data inputs**		

7	Describe and cite additional data inputs	3–4 (Section 2.2.3. Population Data; Section 2.7.2. Rehabilitation Modalities and Utilization Probabilities)
**All data inputs**		
8	Provide data in an extractable format or explain access restrictions	16 (Data Availability Statement)
**Data analysis**		
9	Provide a conceptual overview of the analytical approach	4–6 (Section 2.4. Trend Analysis; Section 2.6. Projection of Stroke-Related YLDs and Rehabilitation Demand to 2030)
10	Describe all analytical steps and statistical models	4–6 (Section 2.4. Trend Analysis; Section 2.7.3. YLD-Equivalent Rehabilitation Workload)
11	Describe model evaluation and selection	4–6 (Section 2.4. Trend Analysis)
12	Report model performance and sensitivity analyses (if conducted)	4–6, 15 (Section 2.7.1. Severity Distribution; Results: Sensitivity Analysis)
13	Describe uncertainty estimation methods	4–6 (Section 2.2.1. GBD 2021 Stroke Estimates; Section 2.6.1. Projection of YLD Rates)
14	State access to analytical/statistical code	Not publicly available (Software described in Section 2.4. Trend Analysis)
**Results and discussion**		
15	Provide published estimates in an extractable format	Tables 1–3 (Section 3. Results; Supplementary Table S4)
16	Report quantitative uncertainty (e.g., 95% UIs)	Tables 1–3; Figures (Section 3. Results)
17	Interpret findings in the context of existing evidence	14–15 (Section 4. Discussion)
18	Discuss limitations and modeling assumptions	15 (Section 4.3. Strengths and Limitations)

Supplementary Table S3. Sensitivity Analysis of Stroke Rehabilitation Demand Under Alternative Severity Distribution and Utilization Probability Assumptions — Saudi Arabia, 2021 Baseline and 2030 Projection.

Panel A. 2021 Baseline (Total Stroke YLDs: 36,245)

Scenario	Severity Distribution			YLD-Equivalent Rehabilitation Workload			
	**Mild (mRS 0–2)**	**Moderate (mRS 3)**	**Severe (mRS 4–5)**	**PT**	**OT**	**SLT**	**MCR**
**Base case (30/50/20%)**	10,873	18,122	7,249	**26,459**	**19,391**	**13,229**	**13,411**
Scenario A: Hemorrhagic-Weighted Severity (ICH + SAH: 20/40/40%; Ischemic: 30/50/20%)							
Scenario A values	9,856	17,105	9,284	26,968	20,154	13,891	14,327
Δ vs. base case	−1,017	−1,017	+2,035	*+1.9%*	*+3.9%*	*+5.0%*	*+6.8%*
Scenario B: Utilization Probability Variation (±15%)							
Upper bound (+15%)	10,873	18,122	7,249	30,428	22,300	15,214	15,422
Lower bound (−15%)	10,873	18,122	7,249	22,490	16,482	11,245	11,399

Panel B. 2030 Projection (Total Stroke YLDs: 40,764)

Scenario	Severity Distribution			YLD-Equivalent Rehabilitation Workload			
	**Mild (mRS 0–2)**	**Moderate (mRS 3)**	**Severe (mRS 4–5)**	**PT**	**OT**	**SLT**	**MCR**
**Base case (30/50/20%)**	12,229	20,382	8,153	**29,758**	**21,809**	**14,879**	**15,083**
Scenario A: Hemorrhagic-Weighted Severity							
Scenario A values	11,085	19,237	10,442	30,330	22,667	15,623	16,113
Δ vs. base case	−1,144	−1,145	+2,289	*+1.9%*	*+3.9%*	*+5.0%*	*+6.8%*
Scenario B: Utilization Probability Variation (±15%)							
Upper bound (+15%)	12,229	20,382	8,153	34,222	25,080	17,111	17,345
Lower bound (−15%)	12,229	20,382	8,153	25,294	18,537	12,647	12,820

Abbreviations: ICH, intracerebral hemorrhage; MCR, multidisciplinary comprehensive rehabilitation; mRS, modified Rankin Scale; OT, occupational therapy; PT, physiotherapy; SAH, subarachnoid hemorrhage; SLT, speech–language therapy; YLD, years lived with disability.

Notes: Base case applies a uniform severity distribution (30% mild, 50% moderate, 20% severe) across all stroke subtypes. Scenario A applies a hemorrhagic-weighted severity distribution (20% mild, 40% moderate, 40% severe) to ICH and SAH, reflecting the higher expected disability burden of hemorrhagic stroke, while retaining the base-case distribution for ischemic stroke. Scenario B uniformly varies modality utilization probabilities by ±15% across all severity levels. In 2021, hemorrhagic subtypes (ICH + SAH) account for 28.1% of total absolute stroke YLDs (10,177 of 36,245); the limited share of hemorrhagic YLDs constrains the impact of Scenario A. The 2030 projection assumes constant subtype proportions (ischemic: 71.9%; hemorrhagic: 28.1%) applied to the projected total YLD count of 40,764 (linear extrapolation of absolute YLD trends from 1990–2021). All values rounded to nearest integer.

Supplementary Table S4. Complete Age-Specific YLD Rates (per 100,000) and Absolute YLD Counts by Stroke Subtype, Sex, and Age Group : Saudi Arabia, 2021.

Note: Due to the extensive dimensionality of the complete dataset (4 subtypes × 3 sexes × 13 age groups × 2 metrics = 312 data points), Supplementary Table S4 is provided as a separate Excel file (Supplementary_Table_S4.xlsx) for readability. The table includes all 12 non-overlapping age groups (10–19, 20–24, 25–29, 30–34, 35–39, 40–44, 45–49, 50–54, 55–59, 60–64, 65–74, 75+ years) plus age-standardized rates. Both point estimates and 95% uncertainty intervals are provided for all cells. GBD 2021 estimates.

1- YLD Rates 2021

Subtype	Sex	Age Group	YLD Rate per 100,000	95% UI Lower	95% UI Upper
Stroke	Both	10-19 years	29.26	20.69	38.56
Stroke	Both	20-24 years	44.57	30.52	61.39
Stroke	Both	25-29 years	56.41	36.86	79.07
Stroke	Both	30-34 years	69.7	48.3	93.91
Stroke	Both	35-39 years	86.91	59.6	118.57
Stroke	Both	40-44 years	113.82	79.1	151.58
Stroke	Both	45-49 years	152.5	105.46	203.3
Stroke	Both	50-54 years	208.59	146.85	274.51
Stroke	Both	55-59 years	270.39	196.26	356.66
Stroke	Both	60-64 years	334.95	241.88	430.39
Stroke	Both	65-74 years	480.8	353.22	618.19
Stroke	Both	75+ years	778.64	570.72	1003.55
Stroke	Both	Age-standardized	129.94	95.28	165.3
Stroke	Male	10-19 years	25.6	17.55	34.4

Stroke	Male	20-24 years	37.95	22.53	56.12
Stroke	Male	25-29 years	47.56	28.95	71.94
Stroke	Male	30-34 years	60.62	39.22	86.24
Stroke	Male	35-39 years	76.57	49.25	109.03
Stroke	Male	40-44 years	102	66.01	144.77
Stroke	Male	45-49 years	139.98	94.73	194.65
Stroke	Male	50-54 years	196.58	136.06	266.63
Stroke	Male	55-59 years	262.33	189.93	350.35
Stroke	Male	60-64 years	336.39	233.79	434.92
Stroke	Male	65-74 years	515.8	374.83	670.86
Stroke	Male	75+ years	907.48	652.59	1198.58
Stroke	Male	Age-standardized	131.4	96.95	168.73
Stroke	Female	10-19 years	33.67	22.74	46.7
Stroke	Female	20-24 years	53.31	34.23	79.93
Stroke	Female	25-29 years	68.59	43.99	99.51
Stroke	Female	30-34 years	83.25	54.82	118.97
Stroke	Female	35-39 years	103.58	68.48	142.73
Stroke	Female	40-44 years	134.14	91.39	186.07
Stroke	Female	45-49 years	173.12	117.01	233.96
Stroke	Female	50-54 years	226.82	155.75	298.6
Stroke	Female	55-59 years	282.16	194.79	372.34
Stroke	Female	60-64 years	332.89	236.61	447.51
Stroke	Female	65-74 years	423.94	306.5	546.41
Stroke	Female	75+ years	582.33	409.74	825.57
Stroke	Female	Age-standardized	127.95	93.42	161.5
Ischemic stroke	Both	10-19 years	18.05	12.08	24.81
Ischemic stroke	Both	20-24 years	28.48	17.48	43.04
Ischemic stroke	Both	25-29 years	36.5	22.61	54.48
Ischemic stroke	Both	30-34 years	45.51	29.6	65.75
Ischemic stroke	Both	35-39 years	56.75	37.21	80
Ischemic stroke	Both	40-44 years	75.43	50.97	101.39
Ischemic stroke	Both	45-49 years	103.44	70.84	139.42
Ischemic stroke	Both	50-54 years	149.29	104.57	197.67
Ischemic stroke	Both	55-59 years	207.2	149.25	272.51
Ischemic stroke	Both	60-64 years	273.55	198.48	352.81
Ischemic stroke	Both	65-74 years	419.84	307.08	539.31
Ischemic stroke	Both	75+ years	703.86	515.88	910.94
Ischemic stroke	Both	Age-standardized	101.4	74.82	128.89
Ischemic stroke	Male	10-19 years	15.44	10.13	21.52
Ischemic stroke	Male	20-24 years	23.51	10.34	38.65
Ischemic stroke	Male	25-29 years	29.9	14.76	49.71
Ischemic stroke	Male	30-34 years	39.01	21.69	60.77
Ischemic stroke	Male	35-39 years	49.74	27.52	75.24

Ischemic stroke	Male	40-44 years	68.15	42.24	102.16
Ischemic stroke	Male	45-49 years	96.12	61.2	139.56
Ischemic stroke	Male	50-54 years	143.55	99.35	197.68
Ischemic stroke	Male	55-59 years	204.71	144.28	276.7
Ischemic stroke	Male	60-64 years	279.04	193.65	364.58
Ischemic stroke	Male	65-74 years	456.19	330.04	594.6
Ischemic stroke	Male	75+ years	842.45	608.47	1123.54
Ischemic stroke	Male	Age-standardized	105.54	77.83	136.34
Ischemic stroke	Female	10-19 years	21.19	12.4	31.55
Ischemic stroke	Female	20-24 years	35.04	18.85	56.83
Ischemic stroke	Female	25-29 years	45.59	24.98	73.06
Ischemic stroke	Female	30-34 years	55.19	32.59	83.57
Ischemic stroke	Female	35-39 years	68.05	41.98	98.45
Ischemic stroke	Female	40-44 years	87.95	55.36	124.86
Ischemic stroke	Female	45-49 years	115.49	74.14	161.41
Ischemic stroke	Female	50-54 years	157.99	104.57	215.32
Ischemic stroke	Female	55-59 years	210.83	145.42	284.25
Ischemic stroke	Female	60-64 years	265.74	185.28	359.87
Ischemic stroke	Female	65-74 years	360.78	259.38	467.08
Ischemic stroke	Female	75+ years	492.68	345.17	708.8
Ischemic stroke	Female	Age-standardized	95.46	70.15	120.14
Intracerebral hemorrhage	Both	10-19 years	7.7	5.39	10.19
Intracerebral hemorrhage	Both	20-24 years	11.29	7.71	16.05
Intracerebral hemorrhage	Both	25-29 years	13.82	8.89	19.96
Intracerebral hemorrhage	Both	30-34 years	16.4	10.7	23.33
Intracerebral hemorrhage	Both	35-39 years	20.42	11.83	30.51
Intracerebral hemorrhage	Both	40-44 years	26.44	16.24	41.49
Intracerebral hemorrhage	Both	45-49 years	35.39	22.18	51.88
Intracerebral hemorrhage	Both	50-54 years	44.5	29.26	63.71
Intracerebral hemorrhage	Both	55-59 years	47.01	30.12	65.5
Intracerebral hemorrhage	Both	60-64 years	44	28.41	62.71
Intracerebral hemorrhage	Both	65-74 years	42.97	29.47	58.94
Intracerebral hemorrhage	Both	75+ years	53.16	35.97	73.05
Intracerebral hemorrhage	Both	Age-standardized	20.22	14.44	26.24
Intracerebral hemorrhage	Male	10-19 years	7.33	5.08	9.69
Intracerebral hemorrhage	Male	20-24 years	10.5	7.33	14.2

Intracerebral hemorrhage	Male	25-29 years	12.66	7.5	19.68
Intracerebral hemorrhage	Male	30-34 years	15.27	9.77	22.39
Intracerebral hemorrhage	Male	35-39 years	18.94	9.23	30.37
Intracerebral hemorrhage	Male	40-44 years	24.27	10.91	43.47
Intracerebral hemorrhage	Male	45-49 years	32.99	16.79	52.01
Intracerebral hemorrhage	Male	50-54 years	41.49	24.83	63.15
Intracerebral hemorrhage	Male	55-59 years	44.78	25.72	65.45
Intracerebral hemorrhage	Male	60-64 years	44.11	25.3	68.47
Intracerebral hemorrhage	Male	65-74 years	46.01	30.48	63.62
Intracerebral hemorrhage	Male	75+ years	51.73	34.71	71.9
Intracerebral hemorrhage	Male	Age-standardized	19.44	13.89	25.32
Intracerebral hemorrhage	Female	10-19 years	8.15	5.67	10.91
Intracerebral hemorrhage	Female	20-24 years	12.32	7.4	20.18
Intracerebral hemorrhage	Female	25-29 years	15.42	9.47	24.53
Intracerebral hemorrhage	Female	30-34 years	18.09	8.63	30.43
Intracerebral hemorrhage	Female	35-39 years	22.8	9.36	38.26
Intracerebral hemorrhage	Female	40-44 years	30.18	16.59	47.47
Intracerebral hemorrhage	Female	45-49 years	39.35	20.95	63.46
Intracerebral hemorrhage	Female	50-54 years	49.07	27.28	74.52
Intracerebral hemorrhage	Female	55-59 years	50.26	31.61	76.19
Intracerebral hemorrhage	Female	60-64 years	43.84	26.33	66.56
Intracerebral hemorrhage	Female	65-74 years	38.05	24.85	52.62
Intracerebral hemorrhage	Female	75+ years	55.32	36.55	80.96
Intracerebral hemorrhage	Female	Age-standardized	21.35	15.11	27.79
Subarachnoid hemorrhage	Both	10-19 years	3.51	2.4	4.73
Subarachnoid hemorrhage	Both	20-24 years	4.8	3.34	6.45
Subarachnoid hemorrhage	Both	25-29 years	6.09	4.13	8.09
Subarachnoid hemorrhage	Both	30-34 years	7.8	5.23	10.22
Subarachnoid hemorrhage	Both	35-39 years	9.74	6.71	13.69

Subarachnoid hemorrhage	Both	40-44 years	11.94	7.63	17.23
Subarachnoid hemorrhage	Both	45-49 years	13.67	8.99	19.7
Subarachnoid hemorrhage	Both	50-54 years	14.8	9.13	22.12
Subarachnoid hemorrhage	Both	55-59 years	16.19	9.86	24.76
Subarachnoid hemorrhage	Both	60-64 years	17.39	10.85	25.16
Subarachnoid hemorrhage	Both	65-74 years	17.98	12.21	24.9
Subarachnoid hemorrhage	Both	75+ years	21.63	13.94	30.92
Subarachnoid hemorrhage	Both	Age-standardized	8.32	5.92	10.91
Subarachnoid hemorrhage	Male	10-19 years	2.83	1.94	3.88
Subarachnoid hemorrhage	Male	20-24 years	3.94	2.65	5.39
Subarachnoid hemorrhage	Male	25-29 years	5	3.38	6.75
Subarachnoid hemorrhage	Male	30-34 years	6.34	4.23	8.53
Subarachnoid hemorrhage	Male	35-39 years	7.89	5.34	10.63
Subarachnoid hemorrhage	Male	40-44 years	9.58	6.54	12.69
Subarachnoid hemorrhage	Male	45-49 years	10.87	7.58	14.57
Subarachnoid hemorrhage	Male	50-54 years	11.54	6.97	17.54
Subarachnoid hemorrhage	Male	55-59 years	12.85	8.01	19.6
Subarachnoid hemorrhage	Male	60-64 years	13.25	8.45	20.23
Subarachnoid hemorrhage	Male	65-74 years	13.6	8.68	19.9
Subarachnoid hemorrhage	Male	75+ years	13.29	8.04	19.81
Subarachnoid hemorrhage	Male	Age-standardized	6.43	4.51	8.54
Subarachnoid hemorrhage	Female	10-19 years	4.33	2.98	5.74
Subarachnoid hemorrhage	Female	20-24 years	5.95	4.06	7.83
Subarachnoid hemorrhage	Female	25-29 years	7.58	5.13	10.26
Subarachnoid hemorrhage	Female	30-34 years	9.96	6.72	13.12
Subarachnoid hemorrhage	Female	35-39 years	12.73	7.99	19.59
Subarachnoid hemorrhage	Female	40-44 years	16.01	7.22	27.89
Subarachnoid hemorrhage	Female	45-49 years	18.29	9.44	29.99
Subarachnoid hemorrhage	Female	50-54 years	19.76	9.92	31.84

Subarachnoid hemorrhage	Female	55-59 years	21.07	10.03	35.76
Subarachnoid hemorrhage	Female	60-64 years	23.31	13.08	37.16
Subarachnoid hemorrhage	Female	65-74 years	25.11	15.64	36.36
Subarachnoid hemorrhage	Female	75+ years	34.32	21.5	51.8
Subarachnoid hemorrhage	Female	Age-standardized	11.14	7.87	14.79

2- Absolute YLDs 2021

Subtype	Sex	Age Group	Absolute YLDs	95% UI Lower	95% UI Upper
Stroke	Both	10-19 years	1491.3	1054.2	1965.3
Stroke	Both	20-24 years	1398.6	957.6	1926.5
Stroke	Both	25-29 years	2154.9	1408.1	3020.1
Stroke	Both	30-34 years	3111.9	2156.2	4192.5
Stroke	Both	35-39 years	3938.4	2701.1	5373.3
Stroke	Both	40-44 years	4434.5	3081.9	5906
Stroke	Both	45-49 years	4394.2	3038.8	5858.1
Stroke	Both	50-54 years	3813.7	2685	5018.9
Stroke	Both	55-59 years	3309.9	2402.4	4365.9
Stroke	Both	60-64 years	2561.5	1849.8	3291.5
Stroke	Both	65-74 years	3746	2752	4816.4
Stroke	Both	75+ years	1889.8	1385.2	2435.7
Stroke	Both	TOTAL	36244.9		
Stroke	Male	10-19 years	712.1	488.1	957
Stroke	Male	20-24 years	677.9	402.4	1002.4
Stroke	Male	25-29 years	1051.6	640.1	1590.9
Stroke	Male	30-34 years	1620	1048	2304.4
Stroke	Male	35-39 years	2142.1	1377.7	3050.2
Stroke	Male	40-44 years	2513	1626.3	3566.7
Stroke	Male	45-49 years	2509.8	1698.3	3489.9
Stroke	Male	50-54 years	2167.5	1500.2	2939.8
Stroke	Male	55-59 years	1906.7	1380.5	2546.4

Stroke	Male	60-64 years	1511.8	1050.7	1954.7
Stroke	Male	65-74 years	2487.4	1807.6	3235.2
Stroke	Male	75+ years	1329.8	956.3	1756.3
Stroke	Male	TOTAL	20629.7		
Stroke	Female	10-19 years	779.3	526.3	1080.9
Stroke	Female	20-24 years	720.7	462.7	1080.6
Stroke	Female	25-29 years	1103.3	707.6	1600.6
Stroke	Female	30-34 years	1491.9	982.4	2132.2
Stroke	Female	35-39 years	1796.3	1187.6	2475.2
Stroke	Female	40-44 years	1921.6	1309.2	2665.4
Stroke	Female	45-49 years	1884.5	1273.7	2546.7
Stroke	Female	50-54 years	1646.3	1130.4	2167.2
Stroke	Female	55-59 years	1403.2	968.6	1851.6
Stroke	Female	60-64 years	1049.7	746.1	1411.2
Stroke	Female	65-74 years	1258.6	909.9	1622.2
Stroke	Female	75+ years	560	394.1	794
Stroke	Female	TOTAL	15615.2		
Ischemic stroke	Both	10-19 years	919.9	615.7	1264.5
Ischemic stroke	Both	20-24 years	893.7	548.5	1350.6
Ischemic stroke	Both	25-29 years	1394.4	863.7	2081.1
Ischemic stroke	Both	30-34 years	2031.6	1321.4	2935.3
Ischemic stroke	Both	35-39 years	2571.6	1686.2	3625.6
Ischemic stroke	Both	40-44 years	2939	1985.9	3950.5
Ischemic stroke	Both	45-49 years	2980.4	2041.1	4017.3
Ischemic stroke	Both	50-54 years	2729.5	1912	3614.2
Ischemic stroke	Both	55-59 years	2536.3	1827	3335.8
Ischemic stroke	Both	60-64 years	2092	1517.9	2698.2
Ischemic stroke	Both	65-74 years	3271	2392.5	4201.8
Ischemic stroke	Both	75+ years	1708.3	1252.1	2210.9
Ischemic stroke	Both	TOTAL	26067.8		

Ischemic stroke	Male	10-19 years	429.4	281.9	598.5
Ischemic stroke	Male	20-24 years	420	184.6	690.3
Ischemic stroke	Male	25-29 years	661.1	326.4	1099.2
Ischemic stroke	Male	30-34 years	1042.5	579.6	1623.8
Ischemic stroke	Male	35-39 years	1391.4	769.9	2104.8
Ischemic stroke	Male	40-44 years	1679.1	1040.6	2517
Ischemic stroke	Male	45-49 years	1723.3	1097.2	2502.1
Ischemic stroke	Male	50-54 years	1582.8	1095.4	2179.6
Ischemic stroke	Male	55-59 years	1487.9	1048.6	2011.1
Ischemic stroke	Male	60-64 years	1254.1	870.3	1638.5
Ischemic stroke	Male	65-74 years	2200	1591.6	2867.4
Ischemic stroke	Male	75+ years	1234.5	891.6	1646.4
Ischemic stroke	Male	TOTAL	15106		
Ischemic stroke	Female	10-19 years	490.4	287	730.2
Ischemic stroke	Female	20-24 years	473.7	254.8	768.2
Ischemic stroke	Female	25-29 years	733.3	401.8	1175.2
Ischemic stroke	Female	30-34 years	989.1	584.1	1497.8
Ischemic stroke	Female	35-39 years	1180.2	728	1707.4
Ischemic stroke	Female	40-44 years	1259.8	793	1788.6
Ischemic stroke	Female	45-49 years	1257.1	807	1757
Ischemic stroke	Female	50-54 years	1146.7	758.9	1562.8
Ischemic stroke	Female	55-59 years	1048.4	723.1	1413.5
Ischemic stroke	Female	60-64 years	838	584.3	1134.8
Ischemic stroke	Female	65-74 years	1071.1	770	1386.7
Ischemic stroke	Female	75+ years	473.8	332	681.7
Ischemic stroke	Female	TOTAL	10961.7		
Intracerebral hemorrhage	Both	10-19 years	392.4	274.8	519.4
Intracerebral hemorrhage	Both	20-24 years	354.2	242	503.7
Intracerebral hemorrhage	Both	25-29 years	528	339.7	762.6
Intracerebral hemorrhage	Both	30-34 years	732.3	477.7	1041.7

Intracerebral hemorrhage	Both	35-39 years	925.2	536.1	1382.8
Intracerebral hemorrhage	Both	40-44 years	1030.2	632.9	1616.5
Intracerebral hemorrhage	Both	45-49 years	1019.8	639	1494.9
Intracerebral hemorrhage	Both	50-54 years	813.6	535	1164.8
Intracerebral hemorrhage	Both	55-59 years	575.4	368.7	801.7
Intracerebral hemorrhage	Both	60-64 years	336.5	217.3	479.6
Intracerebral hemorrhage	Both	65-74 years	334.8	229.6	459.2
Intracerebral hemorrhage	Both	75+ years	129	87.3	177.3
Intracerebral hemorrhage	Both	TOTAL	7171.4		
Intracerebral hemorrhage	Male	10-19 years	203.8	141.4	269.7
Intracerebral hemorrhage	Male	20-24 years	187.6	131	253.6
Intracerebral hemorrhage	Male	25-29 years	279.9	165.8	435.3
Intracerebral hemorrhage	Male	30-34 years	408	261.1	598.3
Intracerebral hemorrhage	Male	35-39 years	529.9	258.3	849.6
Intracerebral hemorrhage	Male	40-44 years	597.8	268.7	1070.9
Intracerebral hemorrhage	Male	45-49 years	591.5	301	932.5
Intracerebral hemorrhage	Male	50-54 years	457.5	273.8	696.3
Intracerebral hemorrhage	Male	55-59 years	325.5	186.9	475.7
Intracerebral hemorrhage	Male	60-64 years	198.2	113.7	307.7
Intracerebral hemorrhage	Male	65-74 years	221.9	147	306.8
Intracerebral hemorrhage	Male	75+ years	75.8	50.9	105.4
Intracerebral hemorrhage	Male	TOTAL	4077.4		
Intracerebral hemorrhage	Female	10-19 years	188.6	131.1	252.5
Intracerebral hemorrhage	Female	20-24 years	166.6	100	272.8
Intracerebral hemorrhage	Female	25-29 years	248.1	152.4	394.5
Intracerebral hemorrhage	Female	30-34 years	324.3	154.6	545.4
Intracerebral hemorrhage	Female	35-39 years	395.3	162.4	663.5
Intracerebral hemorrhage	Female	40-44 years	432.3	237.6	680
Intracerebral hemorrhage	Female	45-49 years	428.3	228	690.7

Intracerebral hemorrhage	Female	50-54 years	356.1	198	540.9
Intracerebral hemorrhage	Female	55-59 years	249.9	157.2	378.9
Intracerebral hemorrhage	Female	60-64 years	138.3	83	209.9
Intracerebral hemorrhage	Female	65-74 years	112.9	73.8	156.2
Intracerebral hemorrhage	Female	75+ years	53.2	35.2	77.9
Intracerebral hemorrhage	Female	TOTAL	3094		
Subarachnoid hemorrhage	Both	10-19 years	179	122.1	241.3
Subarachnoid hemorrhage	Both	20-24 years	150.7	104.7	202.3
Subarachnoid hemorrhage	Both	25-29 years	232.5	157.7	309
Subarachnoid hemorrhage	Both	30-34 years	348	233.4	456.3
Subarachnoid hemorrhage	Both	35-39 years	441.6	304	620.2
Subarachnoid hemorrhage	Both	40-44 years	465.4	297.3	671.3
Subarachnoid hemorrhage	Both	45-49 years	394	259	567.7
Subarachnoid hemorrhage	Both	50-54 years	270.6	167	404.4
Subarachnoid hemorrhage	Both	55-59 years	198.1	120.7	303.1
Subarachnoid hemorrhage	Both	60-64 years	133	83	192.4
Subarachnoid hemorrhage	Both	65-74 years	140.1	95.1	194
Subarachnoid hemorrhage	Both	75+ years	52.5	33.8	75.1
Subarachnoid hemorrhage	Both	TOTAL	3005.7		
Subarachnoid hemorrhage	Male	10-19 years	78.8	54	107.9
Subarachnoid hemorrhage	Male	20-24 years	70.3	47.4	96.4
Subarachnoid hemorrhage	Male	25-29 years	110.6	74.7	149.2
Subarachnoid hemorrhage	Male	30-34 years	169.5	113	228
Subarachnoid hemorrhage	Male	35-39 years	220.9	149.3	297.3
Subarachnoid hemorrhage	Male	40-44 years	236	161.1	312.6
Subarachnoid hemorrhage	Male	45-49 years	194.9	135.9	261.2
Subarachnoid hemorrhage	Male	50-54 years	127.2	76.9	193.4
Subarachnoid hemorrhage	Male	55-59 years	93.4	58.2	142.5
Subarachnoid hemorrhage	Male	60-64 years	59.5	38	90.9

Subarachnoid hemorrhage	Male	65-74 years	65.6	41.9	96
Subarachnoid hemorrhage	Male	75+ years	19.5	11.8	29
Subarachnoid hemorrhage	Male	TOTAL	1446.2		
Subarachnoid hemorrhage	Female	10-19 years	100.2	69	132.9
Subarachnoid hemorrhage	Female	20-24 years	80.4	54.9	105.9
Subarachnoid hemorrhage	Female	25-29 years	121.9	82.5	165.1
Subarachnoid hemorrhage	Female	30-34 years	178.5	120.5	235.1
Subarachnoid hemorrhage	Female	35-39 years	220.7	138.6	339.8
Subarachnoid hemorrhage	Female	40-44 years	229.4	103.4	399.5
Subarachnoid hemorrhage	Female	45-49 years	199	102.7	326.4
Subarachnoid hemorrhage	Female	50-54 years	143.4	72	231.1
Subarachnoid hemorrhage	Female	55-59 years	104.8	49.9	177.8
Subarachnoid hemorrhage	Female	60-64 years	73.5	41.2	117.2
Subarachnoid hemorrhage	Female	65-74 years	74.6	46.4	107.9
Subarachnoid hemorrhage	Female	75+ years	33	20.7	49.8
Subarachnoid hemorrhage	Female	TOTAL	1559.5		

3- ASR Trends 1990-2021

Subtype	Measure	Year	ASR per 100,000	95% UI Lower	95% UI Upper
Stroke	Incidence	1990	166.31	152.04	182.25
Stroke	Incidence	1995	167.39	153.87	183.69
Stroke	Incidence	2000	166.62	153.05	182.17
Stroke	Incidence	2005	164.99	150.48	181.25
Stroke	Incidence	2010	147.92	136.34	160.65
Stroke	Incidence	2015	137.81	126.5	150.73
Stroke	Incidence	2021	130.68	119.37	141.72
Stroke	Prevalence	1990	982.45	932.97	1031.89
Stroke	Prevalence	1995	1003.13	956.2	1047.94
Stroke	Prevalence	2000	1001.33	963.67	1040.76
Stroke	Prevalence	2005	981.29	943.31	1020.47
Stroke	Prevalence	2010	982.89	948.39	1020.96
Stroke	Prevalence	2015	970.08	935.98	1006.47
Stroke	Prevalence	2021	965.17	924.94	1006.4
Stroke	YLLs	1990	3208.96	2507.47	4006.69
Stroke	YLLs	1995	3110.41	2561.61	3679.07
Stroke	YLLs	2000	2888.13	2545.32	3240.89

Stroke	YLLs	2005	2629.64	2343.59	2894.9
Stroke	YLLs	2010	2435.9	2182.55	2689.72
Stroke	YLLs	2015	2109.83	1840.03	2388.72
Stroke	YLLs	2021	1893.4	1556.84	2314.42
Stroke	YLDs	1990	133.48	99.3	170.03
Stroke	YLDs	1995	136.32	100.26	171.1
Stroke	YLDs	2000	136.19	98.73	171.92
Stroke	YLDs	2005	133.53	98.22	168.63
Stroke	YLDs	2010	132.99	97.69	168.95
Stroke	YLDs	2015	131.17	95.87	164.62
Stroke	YLDs	2021	129.94	95.28	165.3
Stroke	DALYs	1990	3342.44	2659.86	4147.99
Stroke	DALYs	1995	3246.74	2708.33	3831.03
Stroke	DALYs	2000	3024.32	2704.85	3381.98
Stroke	DALYs	2005	2763.17	2482.64	3023.82
Stroke	DALYs	2010	2568.89	2322.39	2816.28
Stroke	DALYs	2015	2241	1969.97	2520.49
Stroke	DALYs	2021	2023.34	1687.07	2452.47
Ischemic stroke	Incidence	1990	110.66	96.68	127.09
Ischemic stroke	Incidence	1995	112.55	99.16	128.06
Ischemic stroke	Incidence	2000	115.06	102.08	128.55
Ischemic stroke	Incidence	2005	115.17	100.55	131.46
Ischemic stroke	Incidence	2010	105.8	94.41	118.52
Ischemic stroke	Incidence	2015	100.52	89.32	113.58
Ischemic stroke	Incidence	2021	96	85.29	106.79
Ischemic stroke	Prevalence	1990	740.86	693.44	791.15
Ischemic stroke	Prevalence	1995	762.71	720.39	804.12
Ischemic stroke	Prevalence	2000	762.73	728.15	800.07
Ischemic stroke	Prevalence	2005	750.33	714.73	788.81
Ischemic stroke	Prevalence	2010	762.42	728.63	798.84
Ischemic stroke	Prevalence	2015	763.84	732.24	796.1
Ischemic stroke	Prevalence	2021	763.45	724.12	803.14
Ischemic stroke	YLLs	1990	1680.83	1292.43	2107.27
Ischemic stroke	YLLs	1995	1715.72	1418.72	2019.46
Ischemic stroke	YLLs	2000	1648.34	1464.28	1854.62
Ischemic stroke	YLLs	2005	1523.77	1356.26	1689.86
Ischemic stroke	YLLs	2010	1422.38	1255.61	1586.83
Ischemic stroke	YLLs	2015	1253.87	1088.22	1436.93
Ischemic stroke	YLLs	2021	1139.48	941	1401.77
Ischemic stroke	YLDs	1990	98.91	73.21	125.21
Ischemic stroke	YLDs	1995	102.05	75.2	129.47
Ischemic stroke	YLDs	2000	102.19	74.27	129.03
Ischemic stroke	YLDs	2005	100.65	74.06	127.64
Ischemic stroke	YLDs	2010	101.68	74.51	129.02
Ischemic stroke	YLDs	2015	101.9	75.56	127.97

Ischemic stroke	YLDs	2021	101.4	74.82	128.89
Ischemic stroke	DALYs	1990	1779.74	1408.96	2201.17
Ischemic stroke	DALYs	1995	1817.77	1518.68	2117.92
Ischemic stroke	DALYs	2000	1750.53	1575.61	1956.94
Ischemic stroke	DALYs	2005	1624.43	1461.83	1794.78
Ischemic stroke	DALYs	2010	1524.07	1355.65	1694.89
Ischemic stroke	DALYs	2015	1355.77	1182.34	1533.06
Ischemic stroke	DALYs	2021	1240.87	1040.65	1500.1
Intracerebral hemorrhage	Incidence	1990	49.79	44.52	54.32
Intracerebral hemorrhage	Incidence	1995	49.33	45.28	53.54
Intracerebral hemorrhage	Incidence	2000	46.24	42.61	49.98
Intracerebral hemorrhage	Incidence	2005	44.64	41.28	48.28
Intracerebral hemorrhage	Incidence	2010	37.35	34.79	39.88
Intracerebral hemorrhage	Incidence	2015	32.82	30.5	35.52
Intracerebral hemorrhage	Incidence	2021	30.12	27.64	32.53
Intracerebral hemorrhage	Prevalence	1990	179.42	166.83	193.7
Intracerebral hemorrhage	Prevalence	1995	178.23	167.43	190.12
Intracerebral hemorrhage	Prevalence	2000	175.99	165.63	187.52
Intracerebral hemorrhage	Prevalence	2005	171.87	161.05	184.53
Intracerebral hemorrhage	Prevalence	2010	161.75	150.33	173.78
Intracerebral hemorrhage	Prevalence	2015	148.12	139.77	157.74
Intracerebral hemorrhage	Prevalence	2021	146.08	136.97	157.1
Intracerebral hemorrhage	YLLs	1990	1460.51	1127.83	1856.95
Intracerebral hemorrhage	YLLs	1995	1333.86	1088.69	1619.37
Intracerebral hemorrhage	YLLs	2000	1186.6	1034.85	1354.44
Intracerebral hemorrhage	YLLs	2005	1056.1	934.38	1180.32
Intracerebral hemorrhage	YLLs	2010	961	861.29	1069.81
Intracerebral hemorrhage	YLLs	2015	812.62	690.42	927.96
Intracerebral hemorrhage	YLLs	2021	715.84	576.93	868.25
Intracerebral hemorrhage	YLDs	1990	25.21	17.97	32.46
Intracerebral hemorrhage	YLDs	1995	24.92	17.92	31.87
Intracerebral hemorrhage	YLDs	2000	24.59	17.66	31.92

Intracerebral hemorrhage	YLDs	2005	23.96	17.09	30.77
Intracerebral hemorrhage	YLDs	2010	22.49	16.2	28.57
Intracerebral hemorrhage	YLDs	2015	20.54	14.53	26.73
Intracerebral hemorrhage	YLDs	2021	20.22	14.44	26.24
Intracerebral hemorrhage	DALYs	1990	1485.73	1148.84	1883.08
Intracerebral hemorrhage	DALYs	1995	1358.78	1113.6	1649.76
Intracerebral hemorrhage	DALYs	2000	1211.18	1058.79	1378.47
Intracerebral hemorrhage	DALYs	2005	1080.06	958.37	1204.6
Intracerebral hemorrhage	DALYs	2010	983.48	885.49	1091.92
Intracerebral hemorrhage	DALYs	2015	833.16	710.19	951.24
Intracerebral hemorrhage	DALYs	2021	736.06	597.14	890.62
Subarachnoid hemorrhage	Incidence	1990	5.85	5.11	6.61
Subarachnoid hemorrhage	Incidence	1995	5.51	4.83	6.18
Subarachnoid hemorrhage	Incidence	2000	5.33	4.68	6
Subarachnoid hemorrhage	Incidence	2005	5.18	4.53	5.82
Subarachnoid hemorrhage	Incidence	2010	4.77	4.16	5.37
Subarachnoid hemorrhage	Incidence	2015	4.48	3.87	5.03
Subarachnoid hemorrhage	Incidence	2021	4.57	3.96	5.17
Subarachnoid hemorrhage	Prevalence	1990	66.06	60	72.38
Subarachnoid hemorrhage	Prevalence	1995	66.2	60.85	71.93
Subarachnoid hemorrhage	Prevalence	2000	66.58	61.82	71.58
Subarachnoid hemorrhage	Prevalence	2005	62.74	58.25	67.4
Subarachnoid hemorrhage	Prevalence	2010	62.2	57.57	66.99
Subarachnoid hemorrhage	Prevalence	2015	61.29	56.65	65.83
Subarachnoid hemorrhage	Prevalence	2021	58.7	53.59	63.68
Subarachnoid hemorrhage	YLLs	1990	67.61	47.54	101.02
Subarachnoid hemorrhage	YLLs	1995	60.84	46.01	85.26
Subarachnoid hemorrhage	YLLs	2000	53.19	43.25	66.62
Subarachnoid hemorrhage	YLLs	2005	49.76	41.45	60.5

Subarachnoid hemorrhage	YLLs	2010	52.52	38.2	62.25
Subarachnoid hemorrhage	YLLs	2015	43.34	29.48	54.57
Subarachnoid hemorrhage	YLLs	2021	38.09	25.31	52.2
Subarachnoid hemorrhage	YLDs	1990	9.36	6.56	12.17
Subarachnoid hemorrhage	YLDs	1995	9.35	6.52	12.1
Subarachnoid hemorrhage	YLDs	2000	9.41	6.76	12.11
Subarachnoid hemorrhage	YLDs	2005	8.91	6.41	11.63
Subarachnoid hemorrhage	YLDs	2010	8.82	6.39	11.49
Subarachnoid hemorrhage	YLDs	2015	8.73	6.15	11.26
Subarachnoid hemorrhage	YLDs	2021	8.32	5.92	10.91
Subarachnoid hemorrhage	DALYs	1990	76.97	56.74	110.13
Subarachnoid hemorrhage	DALYs	1995	70.19	54.65	95.16
Subarachnoid hemorrhage	DALYs	2000	62.6	52.24	76.56
Subarachnoid hemorrhage	DALYs	2005	58.68	50.34	69.73
Subarachnoid hemorrhage	DALYs	2010	61.34	46.26	72.39
Subarachnoid hemorrhage	DALYs	2015	52.07	38.01	63.53
Subarachnoid hemorrhage	DALYs	2021	46.41	33.48	59.46

Supplementary Table S5. Age-Standardized Rates and Average Annual Percentage Change (AAPC) by Stroke Subtype, All Measures: Saudi Arabia, 1990 vs. 2021.

Cause	Measure	1990 Rate	2021 Rate	% Change	AAPC %	95% CI Lower	95% CI Upper	p-value
Stroke								
	Incidence	166.3	130.7	−21.4%	−0.86	−1.28	−0.44	0.004
	Prevalence	982.4	965.2	−1.8%	−0.10	−0.20	+0.00	0.056
	YLLs	3,209.0	1,893.4	−41.0%	−1.76	−2.07	−1.45	<0.001
	YLDs	133.5	129.9	−2.7%	−0.13	−0.24	−0.02	0.032
	DALYs	3,342.4	2,023.3	−39.5%	−1.67	−1.97	−1.38	<0.001
Ischemic stroke								
	Incidence	110.7	96.0	−13.3%	−0.52	−0.94	−0.09	0.026

	Prevalence	740.9	763.4	+3.0%	+0.06	−0.03	+0.16	0.156
	YLLs	1,680.8	1,139.5	−32.2%	−1.35	−1.81	−0.90	<0.001
	YLDs	98.9	101.4	+2.5%	+0.05	−0.06	+0.15	0.323
	DALYs	1,779.7	1,240.9	−30.3%	−1.26	−1.69	−0.83	<0.001
ICH								
	Incidence	49.8	30.1	−39.5%	−1.76	−2.29	−1.23	<0.001
	Prevalence	179.4	146.1	−18.6%	−0.75	−1.03	−0.46	0.001
	YLLs	1,460.5	715.8	−51.0%	−2.31	−2.49	−2.13	<0.001
	YLDs	25.2	20.2	−19.8%	−0.79	−1.08	−0.51	<0.001
	DALYs	1,485.7	736.1	−50.5%	−2.28	−2.46	−2.10	<0.001
SAH								
	Incidence	5.9	4.6	−22.0%	−0.88	−1.13	−0.63	<0.001
	Prevalence	66.1	58.7	−11.1%	−0.40	−0.57	−0.24	0.002
	YLLs	67.6	38.1	−43.7%	−1.68	−2.21	−1.15	<0.001
	YLDs	9.4	8.3	−11.1%	−0.39	−0.55	−0.23	0.002
	DALYs	77.0	46.4	−39.7%	−1.49	−1.93	−1.04	<0.001

Abbreviations: AAPC, average annual percentage change; CI, confidence interval; DALYs, disability-adjusted life years; ICH, intracerebral hemorrhage; SAH, subarachnoid hemorrhage; YLDs, years lived with disability; YLLs, years of life lost. All rates are per 100,000 population, age-standardized to the GBD reference population. AAPC derived from log-linear regression on 7 milestone-year observations (1990, 1995, 2000, 2005, 2010, 2015, 2021; df = 5). Both sexes combined.
